# Interactive transcriptome analyses of Northern Wild Rice (*Zizania palustris* L.) and *Bipolaris oryzae* show convoluted communications during the early stages of fungal brown spot development

**DOI:** 10.3389/fpls.2024.1350281

**Published:** 2024-04-26

**Authors:** Claudia V. Castell-Miller, Thomas J.Y. Kono, Ashish Ranjan, Daniel C. Schlatter, Deborah A. Samac, Jennifer A. Kimball

**Affiliations:** ^1^ Department of Plant Pathology, University of Minnesota, Saint Paul, MN, United States; ^2^ Minnesota Supercomputing Institute, University of Minnesota, Saint Paul, MN, United States; ^3^ United States Department of Agriculture, Agricultural Research Service, Plant Science Research Unit, Saint Paul, MN, United States; ^4^ Department of Agronomy and Plant Genetics, University of Minnesota, Saint Paul, MN, United States

**Keywords:** RNA sequencing, disease resistance, innate immunity, virulence, effectors

## Abstract

Fungal diseases, caused mainly by *Bipolaris* spp., are past and current threats to Northern Wild Rice (NWR) grain production and germplasm preservation in both natural and cultivated settings. Genetic resistance against the pathogen is scarce. Toward expanding our understanding of the global gene communications of NWR and *Bipolaris oryzae* interaction, we designed an RNA sequencing study encompassing the first 12 h and 48 h of their encounter. NWR activated numerous plant recognition receptors after pathogen infection, followed by active transcriptional reprogramming of signaling mechanisms driven by Ca^2+^ and its sensors, mitogen-activated protein kinase cascades, activation of an oxidative burst, and phytohormone signaling-bound mechanisms. Several transcription factors associated with plant defense were found to be expressed. Importantly, evidence of diterpenoid phytoalexins, especially phytocassane biosynthesis, among expression of other defense genes was found. In *B. oryzae*, predicted genes associated with pathogenicity including secreted effectors that could target plant defense mechanisms were expressed. This study uncovered the early molecular communication between the NWR–*B. oryzae* pathosystem, which could guide selection for allele-specific genes to boost NWR defenses, and overall aid in the development of more efficient selection methods in NWR breeding through the use of the most virulent fungal isolates.

## 1 Introduction

Northern Wild Rice (*Zizania palustris* L.) (NWR) is a diploid (2n=2x=30) outcrossing, aquatic, C3 Gramineae species that grows in shallow waters of rivers and lakes in North America (Oelke, 2007). The species belongs to the subtribe *Zizaniinae* (Xu et al., 2010), within the tribe *Oryzeae*, distinct from the subtribe *Oryzinae*, containing white rice (*Oryza sativa* L.) (Guo and Ge, 2005). Despite the subtribes diverging 26–30 million years ago, genomic synteny and collinearity between species of *Zizania* and *Oryza* are substantial (Haas et al., 2021; Yan et al., 2022). For example, the genomes of NWR, *Z. latifolia*, and *Oryza* spp., including *O. sativa*, share 14,120 protein-coding orthologous groups (Haas et al., 2021). This is a relevant framework to understand the evolutionary history of the NWR genome and guide research for NWR trait development, such as reduced seed-shattering, since microsyntenic gene blocks for shattering between this species and *O. sativa* have been found (Haas et al., 2021). In addition, microsynteny of genomic areas for genes putatively involved in phytoalexin production is present among NWR, *Z. latifolia*, and *O. sativa* (Yan et al., 2022; Wu et al., 2011; this study), which could facilitate future functional gene studies. In addition, genomic comparisons can lead to a better understanding of vulnerabilities since NWR and *O. sativa* share susceptibility to some of the same pathogens (Lee, 1992; Samac and Castell-Miller, 2023).

**Figure 1:**
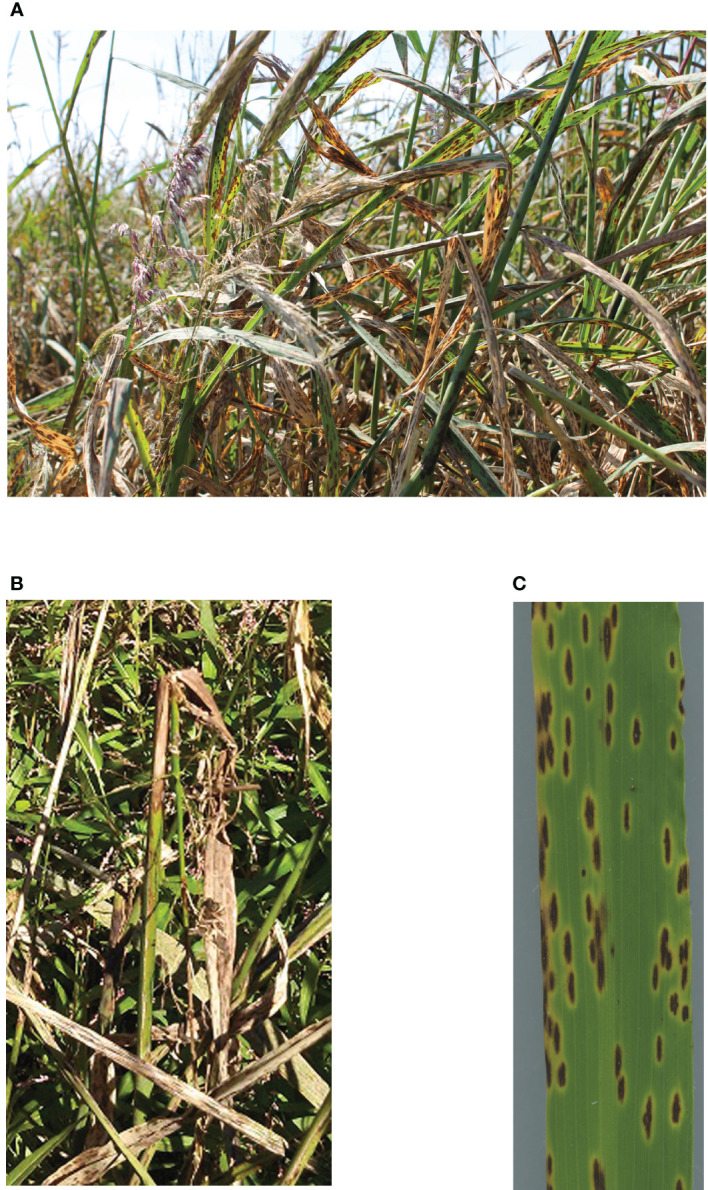
Fungal brown spot symptoms caused by *Bipolaris oryzae* on Northern Wild Rice (*Zizania palustris* L.) plants. **(A)** Overall view of a Northern Wild Rice crop affected by fungal brown spot (FBS) disease. **(B)** FBS symptoms on sheath and stem breakage due to *B. oryzae* infection. **(C)** Fully developed FBS symptoms on leaf.

Since the 1950s, NWR has been commercially cultivated in flooded paddies (Oelke, 2007), where fungal diseases often reduce grain production (Samac and Castell-Miller, 2023). Fungal brown spot (FBS) (Johnson and Percich, 1992), caused by *Bipolaris oryzae* (Breda de Haan) Shoemaker [*Cochliobolus miyabeanus* (S. Ito & Kurib.) Drechsler ex Dastur], is one of the most devastating diseases, causing a serious outbreak in 2015 (Castell-Miller et al., 2021) (**Figure 1A**). *B. oryzae* is mostly considered a necrotrophic fungus (Condon et al., 2013); thus, it presumably kills plant tissue during or soon after invasion (Shao et al., 2021). In artificial inoculations of susceptible NWR cultivars, K2 and Johnson, the fungus has typical bipolar germination by 8 h after inoculation with initial single germ tubes that branch and develop club-shaped appressoria by 24 h. Mostly direct plant cuticle penetration occurs, followed by intra- and intercellular fungal growth. Lesions form between 18 h and 48 h after inoculation (Mitchell Schilkli, 1984). Typical FBS symptoms are purple to brownish spots that enlarge into dark oval or diamond-shaped lesions with tan necrotic centers and chlorotic halos (Johnson and Percich, 1992). FBS can develop on leaves, sheaths, stems, and panicles (**Figures 1B, C**) (Samac and Castell-Miller, 2023). To mitigate disease damage in commercial NWR paddies, an integrated disease management system is followed, based on the management of infected residue, plant nutrition, use of fungicides, and planting of improved disease resistant cultivars (Johnson and Percich, 1992; Porter, 2008; Castell-Miller et al., 2021).

*B*. *oryzae* also causes brown spot (BS) disease in white rice (Lee, 1992), where it can inflict significant grain losses (Sunder et al., 2014). While the genetic mechanisms for FBS resistance in NWR are currently unknown, several quantitative trait loci (QTLs) conferring white rice BS resistance have been identified. These QTLs explain roughly 9% to 23% (Mizobuchi et al., 2016), 23% to 26% (Matsumoto et al., 2017), and 15% to 20% (Ota et al., 2021) of the overall phenotypic variation.

Detection of pathogen-/microbe-/damage-associated molecular patterns (PAMP, MAMP, and DAMP, respectively) by pattern recognition receptors (PRRs) such as receptor-like kinases (RLKs) and receptor-like proteins (RLPs) (Zipfel, 2014; Buendia et al., 2018) results in PAMP-triggered immunity (PTI) (Jones and Dangl, 2006; Zipfel, 2009; Boutrot and Zipfel, 2017). PTI triggers production of reactive oxygen species (ROS), activation of ion channels and mitogen-activated protein kinase (MAPK) cascades, leading to transcriptional reprogramming that produces antimicrobial proteinaceous and non-protein-based molecules (Lo Presti et al., 2015; Saijo et al., 2018). In general, PTI confers quantitative resistance to necrotrophic pathogens lacking host specificity (Liao et al., 2022). PAMPs from necrotrophic fungi include chitin and derivatives, and endo-polygalacturonases (PGs), among other molecules (Wang et al., 2014). Fungi often avoid or overcome PTI by secreting apoplastic and symplastic effectors in a spatial and temporal fashion that can manipulate plant signaling and defense responses (Toruño et al., 2016; Buendia et al., 2018). Effectors are usually perceived through direct or indirect contact (Dodds and Rathjen, 2010) by resistance (R) proteins with conserved intracellular domains usually of the nucleotide-binding leucine-rich receptor (NB-LRR) class (Jones and Dangl, 2006; Lo Presti et al., 2015) activating a highly specific, rapid, and robust response called effector-triggered immunity (ETI) (Jones and Dangl, 2006; Dodds and Rathjen, 2010), often referred to as a hypersensitive reaction (HR). ETI conferring gene-to-gene resistance is an uncommon response to necrotrophic pathogens (Staal et al., 2008), and in some cases, effector perception by R proteins leads to susceptibility to host-specific necrotrophy that exploits plant defenses in an inverse gene-for-gene manner (Faris et al., 2010; Lorang, 2019). Plant defenses against necrotrophic pathogen threats are usually built independently of ETI (Liao et al., 2022).

Overall, effectors of necrotrophic pathogens are phytotoxins, either host selective (HS) or non-HS, including polyketides, non-ribosomal peptides, alkaloids, terpenes, and proteinaceous molecules that can cause cell death, and even induce cell survival during initial plant colonization and manipulate plant defenses (Toruño et al., 2016; Shao et al., 2021). Some HS toxins (HST) from necrotrophic fungi, including *Cochliobolus* spp., can elicit an HR to obtain nutrients from dead tissues (Wang et al., 2014; Liu et al., 2015; Lorang, 2019). Although *B. oryzae* isolates have genomes well-equipped with pathogenicity/virulence genes (Oide et al., 2006; Moriwaki et al., 2007; Condon et al., 2013; Castell-Miller et al., 2016), no HSTs have been found as in other *Cochiobolus* spp (Condon et al., 2013). Non-HST variants of sesquiterpene ophiobolins are produced by *Bipolaris* spp (Xiao et al., 1991) that mainly affect roots and induce stomatal opening (Au et al., 2000). Type A appears to be associated with the virulence of *B. oryzae* on rice (De Bruyne et al., 2016).

Little is known about the molecular basis of innate defenses employed by NWR against plant pathogens. Recently, it was reported that the *Z. latifolia* genome has clusters of genes putatively involved in phytoalexin biosynthesis (Yan et al., 2022).

In this study, we investigated the dual transcriptomes of the NWR cultivar Itasca–C12 (Porter, 2008), and the *B. oryzae* isolate TG12Lb2 (Castell-Miller et al., 2016) in the early stage of disease development since expression profiling at the onset of plant–pathogen interactions will likely identify plant responses associated with perception, defense activation, and fungal pathogenesis pathways (Shen et al., 2017). This decision was also based on observations that in NWR, FBS leaf symptoms develop between 18 h and 30 h in greenhouse conditions, depending on the plant and fungus genotypes used, with clearly visible lesions at 48 h along all the leaf areas inoculated.

This research will increase our understanding of this pathosystem, with the goal of identifying relevant candidate fungal virulence/effector genes for population functional studies, and importantly, it constitutes the baseline to uncover NWR defense responses as a primary step toward functional genomic association studies, development of molecular marker-assisted selection, and potentially pyramiding FBS-resistant alleles.

The specific objectives were to (1) assemble and annotate the non-redundant reference transcriptomes of NWR and *B. oryzae* and (2) identify NWR and *B. oryzae* genes putatively associated with defense and pathogenicity, respectively.

## 2 Materials and methods

### 2.1 Plant material and greenhouse conditions

The NWR cultivar Itasca-C12 with enhanced levels of resistance to foliar diseases including FBS (Porter, 2008) was used in this study. Seeds were germinated in tap water for 7 days at room temperature and ambient light before planting. Three germinated seeds were planted in plastic pots [20.3 cm in diameter × 20.3 cm in height (7.6 L)] containing a pasteurized mixture of topsoil:sand:peat:compost (6:6:5:2 v/v). A 6-g fertilizer tablet (14-4-6; N-P-K; Remke, Remke Enterprise Inc., Downers Grove, IL) was placed on the soil mix after 1/3 of the pot was filled, and a thin layer of 10% iron chelate (Sprint 330, Becker-Underwood Inc., Ames, Iowa) was sprinkled on the soil after 2/3 of the pot was filled and later covered with the soil mix to the top of the pot. The pots were placed into aluminum tanks (66 cm wide × 183 cm long × 70 cm deep) located in a greenhouse at the University of Minnesota (Plant Growth Facility), St. Paul, MN. The tanks were filled with cool water (18°C), 2 to 4 cm above the pot surface. Water was circulated constantly under 5 psi through the tanks to avoid algae formation. Greenhouse air temperature was set at 22°C ( ± 2°C), and each tank was under two 450-W high-pressure sodium halogen lamps supplemented with three 60-W incandescent lights for 16 h/day.

### 2.2 Fungal strain and *in vitro* growth conditions for plant inoculation and RNA extraction

The sequenced strain *B. oryzae* TG12Lb2 (formerly *Cm*TG12bL2; Castell-Miller et al., 2016) was used for plant inoculation and growth *in vitro*. Fungal growth for spore production was done in 2% water agar (BD Bacto™, Becton, Dickinson and Co, Sparks MD, USA) as previously described (Castell-Miller et al., 2016). Spores were collected by adding sterile deionized water to each plate and gently dislodging the spores with an autoclaved rubber policeman spatula. For plant inoculations, spores were diluted as specified in the next section. For transcriptome analysis, that is, to assess gene expression of the fungus grown in artificial media (hereafter “*in vitro*”), 6 mL of spore solution (1.5 × 10^5^/mL) was added to 200 mL of potato dextrose broth (Difco Laboratories, Detroit, MI, USA) in a 500-mL flask. Flasks were shaken at 150 rpm for either 24 h or 48 h at 24°C (± 2°C) in ambient light. There were two replicates of the fungus for each testing time point. Mycelia were harvested by filtering through sterilized cheesecloth, rinsed with sterile deionized water, blotted on sterile filter paper (Whatman), immediately placed in liquid nitrogen, and kept at −80°C until used for RNA extraction.

### 2.3 Plant inoculation and sampling

Plants used for inoculations were between the principal phenological stages (PPS) of stem elongation (PPS3) and booting (PPS4) (Duquette and Kimball, 2020). Leaves of 10 plants were sprayed with 1.5 to 2 mL of spore solution at 15,000 to 20,000 conidia/mL and 0.01% Tween20, while sterile deionized water with Tween20 was sprayed on another 10 plants (hereafter called “mock”). Plants were immediately placed in a mist chamber and received 20 min of continuous mist, followed by 2 min of mist every 60 min during a period of 16 h at 24°C (± 1°C). The plants were allowed to dry slowly and then moved back to the greenhouse. The flag leaf and flag leaf-1 of fungal inoculated and mock treatments were collected at 24 h and 48 h, immediately frozen in liquid nitrogen, and kept at −80°C until used for RNA extraction.

The experiment was set as a completely randomized design with two treatments, fungal and mock inoculated, at two time points of 24 h and 48 h, with three and four biological replicates for each time point, respectively.

### 2.4 RNA extraction and cDNA sequencing

Total RNA was extracted using the RNeasy Mini Kit (Qiagen Inc, Valencia, CA) according to the manufacturer’s instructions. Genomic DNA was removed using the DNA-free™ Kit (ThermoFisher Scientific, Waltham, MA). RNA concentration and integrity, cDNA preparation, and sequencing were done at the Biomedical Genomics Center at the University of Minnesota (https://genomics.umn.edu/). Single pass reads (SP) were sequenced as 50-bp runs either on an Illumina HiSeq2500 (24 h) or a HiSeq2000 (48 h) machine. For each Illumina run, all libraries were equally distributed and multiplexed in two lanes of a flow cell. A total of 20 libraries were sequenced (**Supplementary Table 1**). Two libraries from samples collected at 48 h (**Supplementary Table 1**) were also re-sequenced on Illumina HiSeq2500 to assess technical variation between both instruments, and to ensure that the final assemblies and downstream analyses would be done with compatible reads. The relative expression values obtained from the same library sequenced on different instruments were highly concordant (**Supplementary Data 1**; **Supplementary Figure 1**). The two libraries that served as controls between technologies were removed prior to the differential gene expression (DGE) analysis.

### 2.5 Data analyses

#### 2.5.1 Draft and non-redundant NWR and *B. oryzae* transcriptome assemblies

The bioinformatic workflow steps for obtaining the draft and non-redundant reference transcriptomes of cultivated NWR and of *B. oryzae*, transcript annotations, and DGE analysis are shown in **Supplementary Figure 2**. Intermediate steps and results to obtain the transcriptome draft assemblies t_WRm (mock), t_WRi (prior to separation of plant and fungal transcripts), and t_Boiv (fungal growing *in vitro*) are provided in **Supplementary Data 2** and **Supplementary Tables 2–5**.

To identify and separate plant- and fungal-expressed genes *in planta* in the assembly t_WRi, transcripts were searched against the NCBI nonredundant nucleotide sequence database with BLAST+ version 2.8.1 (https://blast.ncbi.nlm.nih.gov/Blast.cgi) (**Supplementary Figure 2**). Up to five sequences with an *e*-value of 1E-5 were retained for each transcript query. Transcripts that likely originated from Viridiplantae were added to the t_WRm assembly, and those of fungi growing *in planta* (t_Bo_ip) were added to the t_Boiv assembly. The resulting plant and fungal transcriptomes were named t_WRim and t_Boiv_ip, respectively (**Supplementary Figure 2**). Finally, t_Boiv_ip and t_WRim transcriptomes were clustered with CD-HIT version 4.6.1 to cluster redundant sequences (Fu et al., 2012). Sequences were grouped into the same cluster if their lengths were <10% different from the longest sequence of the cluster and if they were at least 95% identical over the aligned portion. t_WRim and t_Boiv_ip constituted the (non-redundant) reference transcriptomes.

#### 2.5.2 Annotation of NWR and *B. oryzae* transcripts

Annotation was done on the longest isoform per gene using Trinotate 3.1.1 (Bryant et al., 2017). For NWR transcript annotations, a set of 16 *Gramineae* species in the Ensembl Plants database (*Aegilops tauschii*, *Brachypodium distachyon*, *Eragrostis tef*, *Hordeum vulgare, Leesia perrieri*, *Oryza barthii, O. glaberrima, O. nivara, O. rufipogon, O. sativa*, *Panicum hallii*, *Saccharum spontaneum, Setaria italica*, *Sorghum bicolor*, *Triticum aestivum*, and *Zea mays*), the draft *Z. latifolia* genome (Yan et al., 2022), and the current annotation of the *Z. palustris* draft genome (Haas et al., 2021) were used. Annotation of fungal transcripts was done using annotated genomes of 24 fungi in the Pleosporales in the Ensembl Fungi database (**Supplementary Table 6**) and from previously annotated *B. oryzae* draft gene sequences (assembly LNFW01000000; Castell-Miller et al., 2016). Trinotate was run with TransDecoder 5.5.0 to search for open reading frames (Haas et al., 2013), RNAmmer 1.2 to search for rRNA genes (Lagesen et al., 2007), HMMER 3.2.1 to identify Pfam domains (Finn et al., 2011), and SignalP 4.1 to identify protein secretion signals (Nielsen, 2017). All assembled transcripts were searched against BLAST+/SwissProt and annotation databases such as eggNOG, Gene Ontology (GO), and Kyoto Encyclopedia of Genes and Genomes (KEGG). All searches were done with an *e*-value threshold of 1E-5.

#### 2.5.3 Data visualization

Visualization of gene expression by library was done with hierarchical clustering of samples using the average root-mean-square of the log_2_ count/million reads as distances between each pair of samples and plotted using the “pheatmap” package in R (https://cran.r-project.org/package=pheatmap). Additionally, reduction of dimensionality and data structure visualization was done using principal components analysis (PCA) on normalized expression values with the “prcomp” function in the R “stats” package.

#### 2.5.4 Differential gene expression analyses

Raw reads of individual libraries (**Supplementary Table 1**) were prepared with Trimmomatic version 0.33. DGE was assessed between plant genes expressed during fungal infection and those expressed in mock-inoculated treatments. Fungal gene expression was estimated between *B. oryzae in planta* and *in vitro* (**Supplementary Figure 2**). Comparisons were made within time points and between treatments. Reads of individual libraries were aligned to either the NWR or the fungal reference non-redundant transcriptomes with Bowtie2 v.2.3.4.1 (Langmead and Salzberg, 2012). Transcript expression was quantified with RSEM 1.3.0 (Li and Dewey, 2011) and transcript-level counts were transformed into gene-level counts using the “tximport” version 1.10.1 package in R (Soneson et al., 2016). Genes with low expression (fewer than three samples with >10 normalized counts) were filtered out prior to dispersion estimations and model fitting, done with the negative binomial generalized linear model implemented in DESeq2 (Love et al., 2014). Shrinkage of the log_2_ (fold change) values was performed with the “apeglm” package in R (Zhu et al., 2019). Genes with a false discovery rate adjusted *p*-value ≤ 0.05 were considered differentially expressed. Volcano plots to visualize changes in gene expression were constructed using the R software, with the log_2_ (fold change) values estimated from the “apeglm” package.

GO enrichment analyses were conducted to assign biological functions to differentially expressed genes (DEGs) at 24 h and 48 h. Hypergeometric tests were used to obtain *p*-values and their adjusted values, *q*-value, based on the Benjamini–Hochberg false discovery rate estimation (Storey and Tibshirani, 2003). The statistically significant threshold was set at *P* = 0.05.

A variancePartition analysis (Hoffman and Schadt, 2016) was carried out to understand the sources of variation in gene expression. A linear random model was used to estimate the proportion of variance explained by time (24 h and 48 h), plant or fungal treatments, and time-by-treatment interaction effects on a gene-by-gene basis. The experimental variables and residuals were considered random effects. Their contributions to variance were estimated with a restricted maximum likelihood (REML) method. The variation partition analysis and the violin graphics with box plots were carried out using the variancePartition R package (Bioconductor: http://bioconductor.org/packages/variancePartition). NWR and fungus genes for which at least 75% of the variation in their expression were explained by treatment were selected for further analysis.

Modules of genes that were highly co-expressed were identified with a weighted gene co-expression network analysis (WGCNA) software (Langfelder and Horvath, 2008) implemented in R. Gene expression in each module was summarized with an eigengene value and tested using an analysis of variance for their association with experimental treatment, time of collection, and treatment-by-time interaction under the model: Eigengene_ijk_ = μ_ijk_ + β_i_ + β_j_ + β_k_ + ε_ijk_, where μ_ijk_ is the average expression value of the genes across samples, β_i_ is the effect of time (24 h or 48 h), β_j_ is the effect of treatment, and β_k_ is the interaction between treatment and time, while ε_ijk_ is the residual variation. Modules were identified as significantly associated with an experimental factor with a Bonferroni correction for multiple hypothesis testing (Benjamini and Hochberg, 1995). The statistical threshold for assessing module significance was P = 0.05, where the number of tests was the number of modules identified in the network analysis. The biological function of genes within a module was resolved using functional KEGG enrichment tests. Hypergeometric tests were used to obtain *p*-values and their adjusted values, *q*-value, based on the Benjamini–Hochberg false discovery rate estimation (Storey and Tibshirani, 2003). The statistical significance threshold was set at P = 0.05.

#### 2.5.5 Selection of candidate genes for plant defense and fungal pathogenesis

Plant genes with annotations potentially involved in defense against *B. oryzae* were identified using an in-house R script on gene-based concatenated data from DEG analyses, selected KEGG enriched pathways within WGCNA-specific modules, and the variancePartition analysis. The terms used in the search are detailed in **Supplementary Data 3**.

Fungal genes related to pathogenesis (**Supplementary Data 3**) were identified from the results of DGE analysis and the variancePartition analyses. In addition, a small subset of secreted proteins containing ≤300 amino acids and ≥4 cysteines were interrogated to predict putative effectors using EffectorP-fungi 3.0 (https://effectorp.csiro.au; Sperschneider and Dodds, 2022) after being filtered for transmembrane domains and N-terminal signal peptides with Phobius (https://phobius.sbc.su.se; Käll et al., 2007).

#### 2.5.6 RT-qPCR gene validation

Gene expression of six NWR genes and five *B. oryzae* genes (**Supplementary Table 7**) was measured for RNA sequencing validation using the RT-qPCR protocol previously described (Castell-Miller et al., 2016). Three NWR genes, elongation factor 1-delta (EF1D), eukaryotic translation initiation factor (IF4D), and glyceraldehyde-3-phosphate dehydrogenase (G3DP), and three *B. oryzae* genes, glyceraldehyde-3-phosphate dehydrogenase (G3DP), ubiquitin-conjugating enzyme (Ubiq), and mitochondrial division protein (MitDiv1), were used as reference genes (RGs) (**Supplementary Table 7**). Statistically significant differences between time points were assessed using a non-parametric test [Wilcoxon sum rank; Chi-square (χ^2^)]. Significance level was set at P < 0.05. RGs were analyzed with RefFinder to identify the most stable plant and fungal gene to use in future studies (Xie et al., 2023). Primers were designed using the PrimerQuest tool (https://www.idtdna.com). Genes and primer sequences are presented in **Supplementary Table 7**.

## 3 Results

### 3.1 Non-redundant NWR and *B. oryzae* transcriptomes and annotations

The reference non-redundant NWR transcriptome t_WRim had a total of 93,610 transcripts and an N50 of 1,656 bp, while that of *B. oryzae*, t_Boiv_ip, had 20,385 transcripts and an N50 of 1,767 bp (**Table 1**).

**Table 1:** Summary statistics for the Northern Wild Rice and *Bipolaris oryzae* transcriptomes collected at 24 h and 48 h after inoculation.

	Transcripts							
Transcriptomes	Number	Length (bp)						N50 (bp)
		Minimum	Median	Mean	Std. Dev	Maximum	Total Assembly	
t_WRim	93,610	251	528	968.9	1,005.0	20,537	90,695,736	1,656
t_Boiv_ip	20,385	251	862	1,182.2	1,006.1	10,502	24,098,438	1,767

WRim = Northern Wild Rice transcriptome with combined t_WRm (mock inoculated plant transcripts) and t_WRi (plant transcripts after separation from those of the fungus growing in planta) at 24 h and 48 h; t_Boiv_ip = Bipolaris oryzae transcriptome with combined transcripts from B. oryzae grown *in vitro* and *in planta* (after separation from plants transcripts in t_WRi) at 24 h and 48 h.

N50: length of the longest contig of all contigs of that length that compose at least 50% of the bases of the assembly.

Approximately 69% of the transcripts within the t_WRim were assigned functional annotations based on sequence similarity to known genes. Within the Ensembl Plants database, the highest matches were annotations to the *Z. palustris* (63.5%), *Z. latifolia* (62.2%), and *O. sativa* (57.2%) genomes, with hits to other grasses varying from 56.8% to 45.6%. Fewer matches corresponded to sequences in the Swiss-Prot database (43.3%); GO categories of Biological Process (BP) (35.1%), Molecular Function (MF) (35.8%), and Cellular Component (CC) (37.2%); Pfam domains (28.8%); and associated KEGG pathways (37.5%). A small percentage of transcripts contained signal peptides (2.5%) (**Supplementary Figure 3**).

Fungal transcripts with annotations (87.7%) matched mostly other *Bipolaris* spp. including *B. oryzae* TG12Lb2 (80.2%) and ATC44560 (82.5%) genes, and fewer sequences at the Swiss-Prot (50.4%) and GO terms in BP (40.2%), MF (43.6%), and CC (41.7%) categories. A little over half had Pfam domains (54.8%), and associated KEGG pathways (39.8%), while a small percentage of transcripts was predicted to be secreted (4.7%) (**Supplementary Figure 3**).

### 3.2 NWR and *B. oryzae* visualization and gene expression analyses

**Figure 2:**
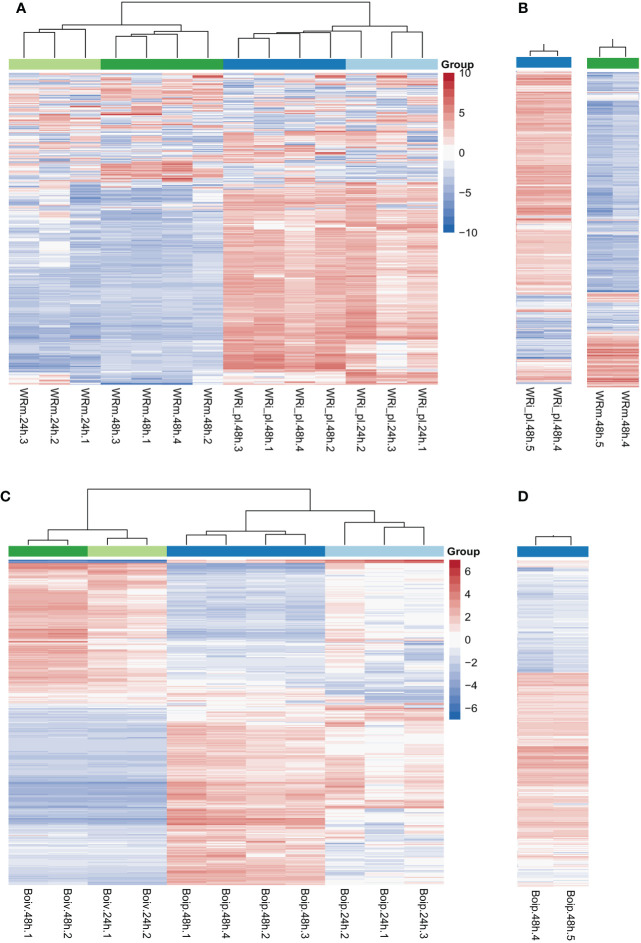
Northern Wild Rice (*Zizania palustris* L.) and *Bipolaris oryzae* clustered heatmaps of relative gene expression. **(A)** Northern Wild Rice (NWR) relative expression after fungal infection (WRi_pl) and mock inoculation (WRm). **(B)** NWR technical replicates of relative expression in WRi_pl (left) and in WRm (right). **(C)** *B. oryzae* relative expression of the fungus grown *in planta* (Boip) and *in vitro* (Boiv). **(D)** *B. oryzae* technical replicates of relative expression in Boip. Time points were 24 h and 48 h = collection hours after mock or fungal inoculation. Number of biological replicates were 1 to 4; 5 = technical replicate of biological replicate 4 for testing consistency between HiSeq2000 and HiSeq2500 Illumina technologies. “Group” colors correspond to the treatment and time point of the sample. The color ramp shows the mean-centered relative expression for each gene. Light red = upregulation relative to mean expression; light blue = downregulation relative to mean expression; white = mean expression. The 500 most variable genes are shown.

Heatmaps showed that the NWR libraries were separated into two distinct clusters based on expression patterns of mock- and fungal-inoculated treatments (**Figure 2A**), and within each of them, they were grouped by time points of collection except for a library of fungal inoculated plants at 24 h that clustered with those of the same treatment at 48 h. Over half of the genes in the mock-inoculated set exhibited low expression or downregulation, while mostly the opposite was observed for roughly the same genes in the fungal-inoculated plant libraries (WRi_pl). Technical replicates for comparing Illumina technologies were similar (**Figure 2B**).

The *B. oryzae* heatmaps showed transcripts separated into two distinct clusters based on *in vitro* and *in planta* growth (**Figure 2C**). There were few differences in gene expression between 24 h and 48 h of *in vitro* growth but there were distinct changes between 24 h and 48 h of *in planta* growth with some variation by biological replicate in the 24-h libraries (**Figure 2C**). The two technical replicates for Illumina technology comparison were similar (**Figure 2D**).

Three principal components (PCs) explained 54.7% of the total variation in gene expression in the NWR data sets (**Supplementary Figure 4A**). The first vector, PC1, accounted for 32.2% and corresponded strongly with mock- vs. fungal-infected treatments. PC2 explained 14.5% and corresponded with time points, and PC3 accounted for 8.0%. Overall, the combinations of these three PCs separated the treatments and time points, except for one library of mock inoculation that grouped with those of fungal inoculated at 24 h.

In the *B. oryzae* data, the PC analysis explained 66.8% (PC1 = 28.4%; PC2 = 25%, and PC3 = 13.4%) of the variation (**Supplementary Figure 4B**). PC1 divided transcripts expressed *in planta* from those *in vitro*, while PC2, in its interaction with PC1, roughly divided transcripts expressed at 24 h from those at 48 h. PC3 (13.4%) together with PC1 contributes to the distinction of *in vitro* and *in vivo* treatments.

**Figure 3:**
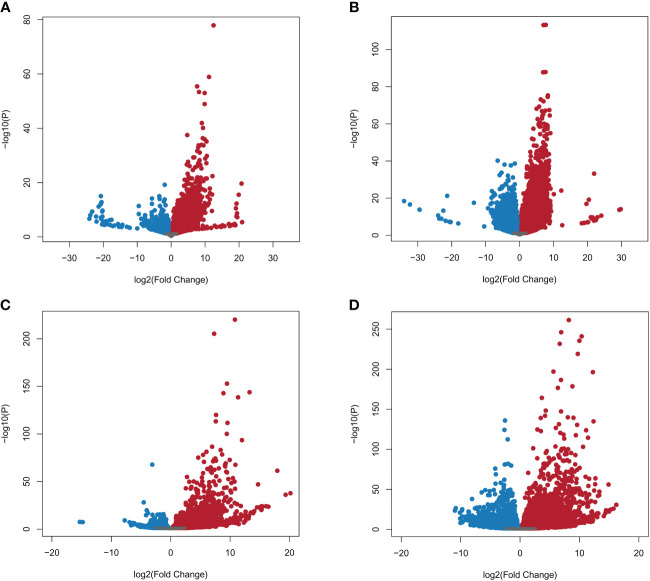
Volcano plots of Northern Wild Rice (*Zizania palustris* L.) and *Bipolaris oryzae* expressed genes by time point. **(A, B)** Log_2_ fold changes of expressed NWR genes in fungal-infected compared to mock-inoculated plants. **(C, D)** Log_2_ fold change of expressed *B. oryzae* genes from *in planta* compared to *in vitro* growth treatments. Time points: **(A, C)** = 24 h, **(B, D)** = 48 h. Gray dots = not differentially expressed genes (DEGs); red dots = upregulated DEGs; blue dots = downregulated DEGs. The statistical significance threshold was set at P = 0.05.

A total of 39,180 NWR genes were differentially expressed, with the numbers of DEGs increasing over time from 10,787 at 24 h to 28,393 at 48 h. At each time point, the total number of upregulated genes was higher than those downregulated (**Table 2**; **Figures 3A, B**). There were more unique genes downregulated than upregulated by time point (**Table 2**). Out of a total of 7,184 genes commonly expressed at both time points, 67.9% were upregulated and 32.1% were downregulated (**Table 2**; **Supplementary Figure 5A**).

**Table 2:** Number of differentially expressed genes in Northern Wild Rice and *Bipolaris oryzae* at 24 h and 48 h after inoculation.

	DGE^a^		
Wild rice	24 h	48 h	24 + 48 h ^b^
Up Down	6,274 (1,340) ^c^ 4,513 (2,143)	14,913 (9,977) 13,480 (11,112)	4,875 2,309
Total	10,787 (3,483)	28,393 (21,089)	7,184
*Bipolaris oryzae*	24 h	48 h	24 + 48 h ^b^
Up Down	1,870 (326) 911 (257)	3,604 (2,057) 2,847 (2,196)	1,513 620
Total	2,781	6,451	2,133

a DGE = differential gene expression.

b Commonly differentially expressed genes at both time points.

c Numbers in parenthesis = unique genes differentially expressed at each time point.

A total of 9,232 *B. oryzae* genes were differentially expressed *in planta* with 2,781 at 24 h and 6,451 at 48 h when compared to transcripts from *in vitro* growth (**Table 2**). A higher number of up- than downregulated genes were found at both collection times (**Table 2**; **Figures 3C, D**). More unique genes were upregulated at 24 h than those downregulated. In addition, 2,133 DEGs were common to both time points with 70.9% up- and 29.1% downregulated genes (**Table 2**; **Supplementary Figure 5B**).

The GO terms assigned to up- and downregulated DEGs were used to infer their biological functions. A total of 53.9% of NWR transcripts had GO annotation terms in at least one of the three categories at 24 h and 52.4% at 48 h. Many of the upregulated NWR DEGs were associated with defenses: defense response (e.g., GO:0006952), response to fungus (e.g., GO:0050832), response to bacterium (e.g., GO:0042742), response to oomycetes (e.g., GO:0002229), diterpene phytoalexin biosynthetic process (e.g., GO:0051502), response to salicylate (e.g., GO:0009751), ethylene-activated signaling pathway (e.g., GO:0009873), response to chitin (e.g., GO:0010200), and response to oxidative stress (e.g., GO:0034599), among others, indicating activation of defense mechanisms. However, there were no statistically supported GO enrichments at both time points.

In the pathogen, 67.6% of DEGs at 24 h and 62.6% of DEGs at 48 h had GO terms in at least one of the three categories. Examples of genes with specific GO terms with links to causing disease were modulation by symbiont of host process (GO:0044003) (that replaced “pathogenesis”; GO:0009405), aflatoxin (GO:0045122), and modulation by symbiont of host defense-related programmed cell death (GO:0034053).

There were 44 (24 h) and 114 (48 h) GO enrichments of upregulated (63) and downregulated (95) fungal genes over the three GO categories. At 24 h, most of the enrichments corresponded to upregulated DEGs, while at 48 h, a majority of the enriched GO terms were from downregulation. Some enrichments found within upregulated DEGs included catabolic and/or metabolic processes of carbohydrates (e.g., GO:0005975; BP) and other molecules, hydrolytic activities (e.g., GO:0016798; MF), carbohydrate binding (GO:0030246; MF), and interaction with the host cell (e.g., GO:0043657; CC). Interestingly, the enriched GO terms associated with the host increased substantially by 48 h (**Supplementary Table 8**).

**Figure 4:**
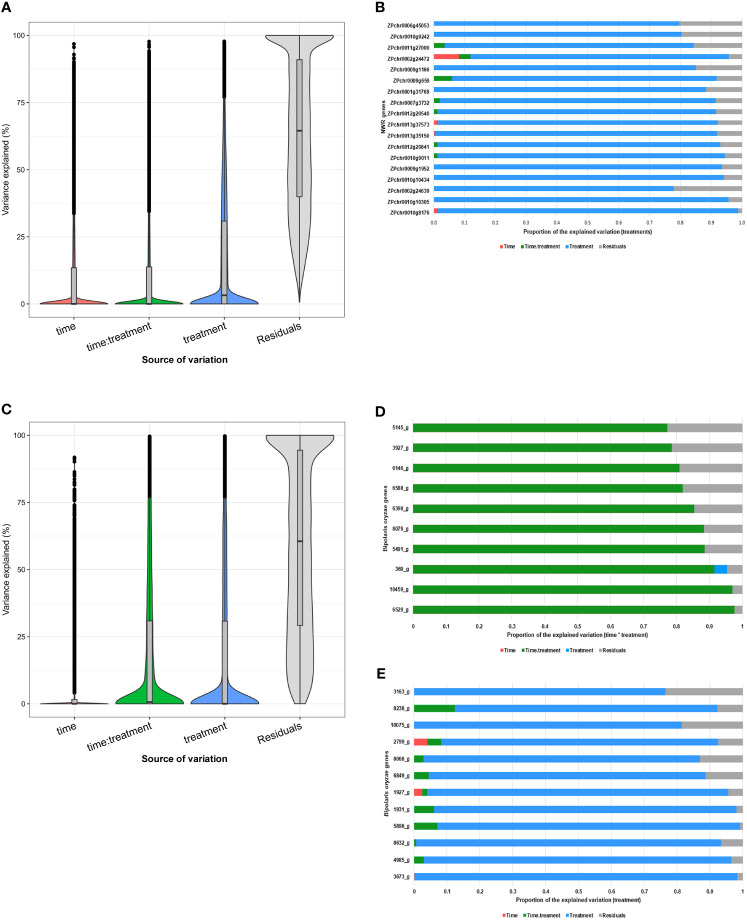
variancePartition analysis of differentially expressed genes in the early interaction between Northern Wild Rice (*Zizania palustris* L.) and *Bipolaris oryzae*. **(A, C)** Violin plots and box plots of log-normal genome-wide gene expression summaries of variance partition contributor variables. **(B, E)** Representative genes with at least 75% of variance explained due to treatment effects and **(D)** due to time by treatment interaction effects. **(A, B)** Northern Wild Rice (NWR); **(C**–**E)** *B. oryzae*. Putative gene annotations are provided in **Supplementary Table 13** (Section 3.3) and **Supplementary Table 14** (Section 3.4). The statistical significance threshold was set at P = 0.05. Source of variation: Time = 24 h and 48 h; treatments = NWR gene expression of fungal-infected vs. mock-inoculated plants and *B oryzae* grown *in planta* vs. *in vitro*; time. treatment = time by treatment interaction, residual = uncharacterized variation. Box plots: 1st horizontal (down) bar = 1st quartile (1.5 interquartile range); 2nd horizontal bar = median; 3rd horizontal bar = 3rd quartile (1.5 interquartile range). Vertical lines indicate 95% confident intervals, dots indicate outliers.

The variancePartition analysis revealed that the main source of NWR gene expression variation was due to individuals (median of 64.5%) and a small effect was due to treatments (median of 3.2%). Time of sampling and its interaction with treatments had a very low effect (**Figure 4A**). A total of 1,456 transcripts explained at least 75% of the effects due to treatments, with many genes associated with defense including PRRs, ROS, Ca^2+^ signaling, transcription factors (TFs), and phytoalexin biosynthesis (**Figure 4B**). In the fungal transcriptome, most of the expression variation was also due to individuals (median of 60.5%). Treatment-by-time interaction explained a small fraction (median = 0.0065%), while time and treatments each had a median of zero (**Figure 4C**). In the interaction between time and treatment, some of the genes were associated with GO terms of modulation by symbiont of host process, catabolism of cellulose and xylan (GO:0030245, GO:0045493), positive regulation of transcription (GO:0045944), and the glyoxylate cycle (GO:0006097) (**Figure 4D**). Despite the small effect of treatments on the global study variation (median = 0%; mean = 17.3%), 669 transcripts drove at least 75% of that variation. In this group, upregulated genes have associated GO terms to modulation by symbiont of host process, the glyoxylate cycle, cutin, cellulose, and xylan catabolic processes, among others (**Figure 4E**).

**Figure 5:**
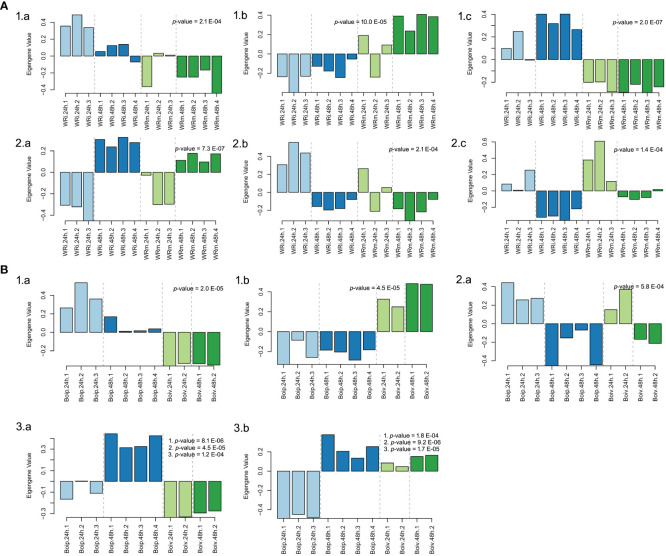
Biological arrangements of statistically significantly different modules in Weighted Gene Co-expression Analyses. **(A)** Northern Wild Rice (*Zizania palustris* L.). (1) Treatment (a = “Aquamarine”, b = “Goldenrod3”, c = “Firebrick4”); (2) Time (a = “Darkmagenta”, b = “Floralwhite”, c = “Lightpink4”). **(B)** *Bipolaris oryzae*. (1) Treatment (a = “lightblue3”, b = “darkviolet”); (2) Time (a = “yellowgreen”); (3) Treatment, time, and time by treatment interaction (a = “darkorange”, b = “darkolivegreen4”). *p*-values correspond to the module’s association to time, treatment, and to time, treatment, and time by treatment interaction as calculated by one-way ANOVA. Numbered *p*-values in panel insets correspond to the following tests: (1) treatment; (2) time; (3) treatment-by-time interaction. The statistical significance threshold was set at P = 0.05.

In the NWR data, a total of 57 gene-expressed modules (**Supplementary Table 9**) were found with the WGCNA. Of those, only three gene modules were statistically significantly associated with treatments: “goldenrod3” (30,954 genes), “aquamarine” (5,312 genes), and “firebrick4” (15,656 genes), while three other modules were statistically linked to the time of sampling (**Figure 5**; **Supplementary Table 9**). Biological functions of genes in statistically supported modules were inferred by enrichment KEGG tests. All modules except “floralwhite” contained several significantly enriched KEGG pathways. Some modules within treatments contained enriched KEGG pathways that could be related to defense mechanisms, such as the “firebrick4”, which contained the pathways plant hormone signal transduction (osa04075), MAPK signaling pathway–plant (osa04016), and plant–pathogen interaction (osa04626). Within the latter pathway, we identified DEGs with GO terms of defense response (GO:0006952), calcium-mediated signaling (GO:0019722), abscisic acid-activated signaling pathway (GO:0009738), intracellular signal transduction (GO:0035556), and activation of protein kinases (GO:0032147), among many others (data not shown). The two WGCNA plant modules statistically associated with time had enriched pathways involved in transcription, translation, autophagy, and circadian rhythm and signaling, among others (**Supplementary Table 10**).

In the fungal network, 20 gene modules were found but only 2 were uniquely associated with the variable treatment, “lightblue3” (48 genes) and “darkviolet” (2,546 genes), while the gene expression profiles in the “yellowgreen” module (374 genes) were statistically associated with time (**Figure 5**). Another two modules, “darkorange” (2,561 genes) and “darkolivegreen4” (1,901), had significant profile expressions associated with time, treatment, and its interaction (**Supplementary Table 11**; **Figure 5**). Enriched pathways were only found in gene expression profiles of “yellowgreen” and “darkorange” modules and were related to carbohydrate metabolism and to translation (**Supplementary Table 12**).

### 3.3 Candidate transcripts associated with plant defenses

Many plant transcripts from upregulated DEGs matched membrane or cytoplasmic receptors with putative roles in defense against microorganisms, including fungi (**Supplementary Table 13**). For example, a few had similarity to wall-associated receptor kinases (WAKs) (DN91548_c0_g1, and DN80941_c0_g1) (Kohorn, 2015), to the LysM Chitin-Elicitor Receptor Kinase 1, CERK1 (DN38604_c0_g1), and the Chitin Elicitor - Binding Protein, CEBiP (DN10871_c0_g1) (Shimizu et al., 2010). A few have multifaceted biological functions in addition to response to diverse stresses. For example, transcript DN27975_c0_g1 matched a leucine-rich repeat receptor kinase with homology to RLK SERK2 involved in somatic embryogenesis, which could mediate defense signaling (Hu et al., 2005). Transcript DN10576_c0_g1 matched the receptor-like protein kinase FERONIA involved in monitoring cell wall integrity (Ji et al., 2020), cell growth, and pathogen invasion (Zhang et al., 2020). Transcript DN12795_c0_g1 matched a membrane glycoprotein receptor, an ethylene-inducing xylanase EIX2 receptor (Ron and Avni, 2004), and several L-type lectin-domain containing receptor kinases (e.g., DN670_c0_g1) with potential roles in plant immunity (Wang and Bouwmeester, 2017). Other examples of expressed receptors are shown in **Supplementary Table 13**.

NWR transcripts also matched respiratory burst oxidase homolog (RBOH) proteins, one of the primary sources of ROS (Dumanović et al., 2021), such as RBOHAs (DN2791_c1_g1; and DN24126_c0_g2) and RBOHB (DN54772_c0_g1), and several extracellular, membrane-bound, or secreted peroxidases (DN26307_c0_g1) and a catalase (DN1935_c0_g2) that could act as detoxifying agents (Camejo et al., 2016). Transcripts with similarity to ROS stress-related proteins such as the receptor-like protein kinase 7 (DN8307_c0_g1) with a role in oxidative stress tolerance (Pitorre et al., 2010) and an oxidative burst-mediated signaling serine/threonine-protein kinase OXI1 (DN52354_c0_g1) (Rentel et al., 2004) were also among the DEGs. Other examples with potential functions in cell protection against oxidative stress spikes were a putative glutathione peroxidase (DN83986_c0_g1), a peroxiredoxin (DN12510_c0_g1), and a probable phospholipid hydroperoxide glutathione peroxidase (DN37333_c1_g1) (**Supplementary Table 13**).

Several transcripts could be associated with calcium ion (Ca^2+^) signaling influencing many biological processes, including activation of early defense mechanisms (Cheval et al., 2013; Zhang et al., 2014). Upregulated DEGs potentially involved in earlier steps of Ca^2+^ signature included those participating in Ca^2+^ channel activity and homeostasis such as a putative homolog of the glutamate-gated receptor (e.g., DN90621_c0_g1) and a cyclic nucleotide-gated ion channel (e.g., DN12940_c0_g1). Other transcripts matched sensors capturing Ca^2+^ signature changes such as calmodulin (CaM) 2/4 (DN12206_c0_g1), and several calmodulin-like proteins (CML) (e.g., DN31307_c0_g1), likely calcium/calmodulin-binding proteins (e.g., DN74167_c0_g1), and a calmodulin-binding protein 60 D (DN24375_c0_g1). Others were similar to Ca^2+^ sensors such as the calcium-dependent proteins kinases (CDPKs) (DN5320_c0_g1 and DN2192_c1_g2), the calmodulin-binding receptor kinase CaMRLK (DN347_c0_g1), CBL-interacting protein kinases (e.g., DN33910_c0_g1), MLO-like (DN1706_c0_g1), Elicitor Protein1 (DN37290_c0_g1), and others shown in **Supplementary Table 13**.

MAPKs play several biological functions in the regulation of plant development, phytohormone signal transduction, and responses to diverse stresses (Chen et al., 2021). Ten out of 15 transcripts with similarity to MAPKs were found in DEGs. Two of those, a putative M2K5 (DN44524_c0_g1) and an MK12 (DN4590_c0_g1), were associated with defense responses, three (DN12577_c0_g1, DN618_c0_g1, and DN87055_c0_g1) were associated with intracellular signal transduction and regulation, and four were associated with abscisic acid activation (DN61222_c0_g1, DN19401_c0_g1, DN19401_c1_g1, and DN15154_c0_g1). DN44524_c0_g1, involved in defenses, was also associated with abscisic acid activation. Others were associated with diverse stress responses and signaling pathways (**Supplementary Table 13**).

TFs are transcriptional activator or repressor proteins that modulate diverse cellular functions by regulating gene expression, including those in PTI, ETI, hormone signaling, and secondary metabolite synthesis (Seo and Choi, 2015). Some representative DEG transcripts with similarity to TFs were the Ethylene-responsive transcription (ERF) factor (DN71940_c0_g1, DN19616_c0_g1), NAM, ATAF1/2, and CUC2 (NAC) family containing proteins (DN3475_c0_g1), WRKY (DN21530_c0_g1 and DN41602_c0_g1), jasmonic acid myeloblastosis viral oncogene homolog (JAMYB) (DN89794_c0_g1), basic leucine zipper transcription factor TGAL (DN81148_c0_g1 and DN19181_c0_g1), basic helix–loop–helix protein (DN25752_c0_g1.p1), and a pathogenesis-related gene transcriptional activator PTI5 (DN41503_c0_g1) (**Supplementary Table 13**).

Phytohormones are regulatory molecules that mediate signaling pathways of diverse biological processes such as plant immune responses to pathogens (Checker et al., 2018). Some upregulated DEGs matched those of the jasmonic acid (JA), salicylic acid (SA), abscisic acid (ABA), and ethylene (ET) biosynthesis and signaling. Examples of transcripts putatively linked to SA signaling were those matching the SA receptor non-expresser NPR1 (DN54187_c0_g1) and the repressor homolog 1 NRR (DN74813_c0_g1) of NPR1 (**Supplementary Table 13**). Others were potentially associated with JA such as lipoxygenases (DN4116_c1_g2) and allene oxide synthase (DN30846_c0_g1), a JA receptor, coronatine-insensitive protein homolog 1a (DN35451_c0_g1), pathway repressors (DN64599_c0_g1), activator MYC2 (DN37456_c0_g1), and other regulatory proteins (**Supplementary Table 13**). Additional transcripts matched ET biosynthesis (1-aminocyclopropane-1-carboxylate oxidase and synthase (DN90223_c0_g1; DN17193_c0_g1) and several TFs associated with ET signaling pathways and defense (**Supplementary Table 13**). The hormone ABA is an essential player in rice signaling defenses against *B. oryzae* (De Vleesschauwer et al., 2010, 2014). Associated with ABA were transcripts similar to the RLPK FERONIA (e.g., DN71845_c0_g1), a positive regulator of ABA signaling of the Exocyst complex component EXO70B1 (DN94177_c0_g1), MAPKs (e.g., DN44524_c0_g1), and associated TFs (e.g., DN16018_c0_g1), among others (**Supplementary Table 13**).

Putative phytohormone DEGs from the brassinosteroid, auxin, and cytokinin-mediated signaling pathways that could also participate in defenses against *B. oryzae* were found (De Vleesschauwer et al., 2010, 2014; Checker et al., 2018).

Several other upregulated DEGs presumably linked to defenses were disease resistance proteins (DN20125_c0_g1 and DN18160_c0_g1) and several pathogenesis-related proteins (PRPs) such as PRMS (DN784_c0_g1), major pollen allergen Bet v 1-D/H (DN9558_c0_g1), zeamatin (DN1913_c0_g1), thaumatin-like (DN17131_c0_g1), osmotin-like protein (DN11744_c0_g1), chitinases (e.g., DN5450_c0_g1), xylanase (e.g., DN6028_c1_g1), and cysteine protease inhibitors (DN6854_c0_g1) (**Supplementary Table 13**).

**Figure 6:**
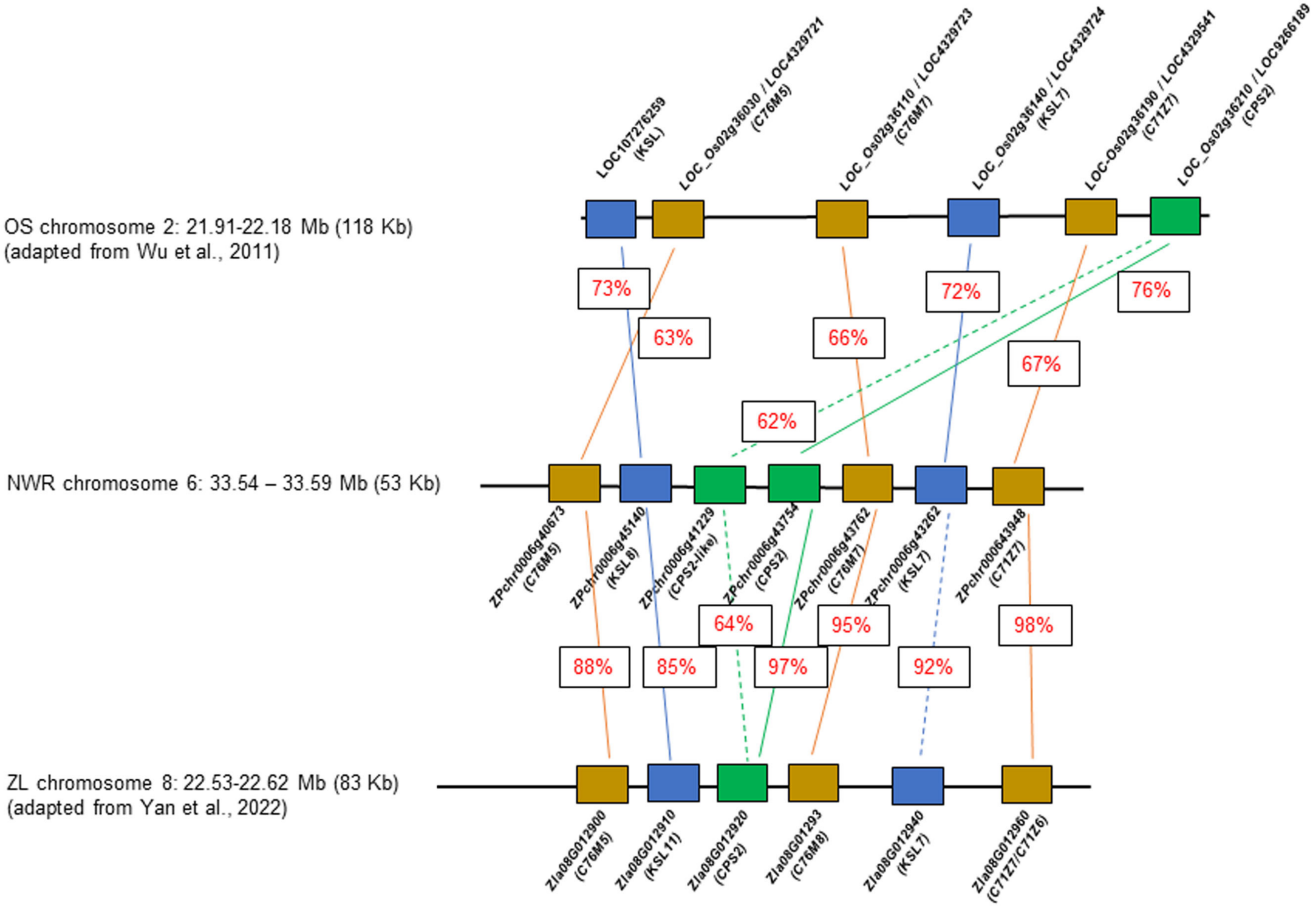
Comparative diagram of the phytoalexin gene cluster between *Zizania palustris* chromosome 6 and *Zizania latifolia* chromosome 8, and with *Oryza sativa* chromosome 2. NWR = Northern Wild Rice (*Zizania palustris* L.); ZL = *Zizania latifolia* L.; OS = *Oryza sativa* L. Putative C76M5, 7, 8; C71Z7, C71Z6 = cytochrome P450 monooxygenases; KSL 7, 8, and 11 = kaurene synthases, CPS2 = ent-copalyl diphosphate synthase 2. Percentage within boxes indicates percent of peptide identity between NWR and ZP and NWR with OS proteins. Brown box = cytochrome P450 monooxygenases; blue box = kaurene synthases; green box = ent-copalyl diphosphate synthase 2.

A few NWR transcripts had similarity to genes for phytoalexin production, likely phytocassanes including two putative copies of class II diterpene synthases, the ent-copalyl diphosphate synthase 2, CPS2 (hereafter denominated CPS2-like; DN7627_c0_g1, ZPchr0006g41229), and CPS2 (DN723_c0_g1; ZPchr0006g43754), an ent-cassadiene C11-alpha-hydroxylase 1 (DN4445_c0_g1, ZPchr0006g43762), a cytochrome P450 76M7 (DN4445_c1_g1, ZPchr0006g43762), an ent-cassa-12,15-diene synthase (DN1038_c1_g1, ZPchr0006g43262), and an ent-cassadiene hydroxylase (DN15743_c0_g1, ZPchr0006g43948). In addition, two other DEGs found in close proximity included a cytochrome P450 76M5 (DN4445_c1_g1, ZPchr0006g40673) and a transcript matching the stemar-13-ene synthase (DN69815_c0_g1, ZPchr0006g45140) potentially involved in oryzalexin synthesis. These seven genes were co-located in ~58,202 bp in chromosome 6 [phytoalexin biosynthetic gene cluster (PBGC)], each separated by an average distance of 5,430 bp (scaffold 48; Haas et al., 2021) (**Figure 6**). PBGC shows microsynteny and collinearity with the corresponding PBGC of *Z. latifolia* on chromosome 8 (Yan et al., 2022), and less to that of white rice (Wu et al., 2011). Additional DEGs matching a momilactone A synthase (DN5222_c0_g1, ZPchr0013g37573) and an ent-isokaurene C2/C3-hydroxylase (DN74967_c0_g1, ZPchr0001g32242) were found and, thus, potentially involved in momilactone and oryzalide biosynthesis (Wu et al., 2011).

### 3.4 Candidate fungal transcripts associated with pathogenesis

A total of 69 peptides without transmembrane motifs and with signal peptides were predicted to be putative effectors (Sperschneider and Dodds, 2022), of which 38 were identified as apoplastic, 19 were identified as cytoplasmic, and a fewer number were predicted to have dual localization, either apoplastic/cytoplasmic (11) or cytoplasmic/apoplastic (1). Ten peptides did not match fungal annotations. Some examples are shown in **Supplementary Table 14**. Additional transcripts with association to pathogenesis terms of necrotrophic fungi included a transcript (DN46467_c0_g1, 4985_g) with high similarity to a putative isocitrate lyase of the pathogenicity ICL1 gene (Q86ZF1) of *Leptosphaeria maculans* (Idnurm and Howlett, 2002). Transcript DN6010_c0_g1 (2994_g) had similarity to the secreted isochorismatase effector ICS1 (G2X4M1.1) of *Verticillum dahliae* (Liu et al., 2014). DN25113_c0_g1 (6024_g) was similar to a necrosis- and ethylene-inducing protein-like NLP effector protein 10 (L7NCS1) of *Phytophthora capsici* (Feng et al., 2014). One nonribosomal peptide synthetase (NPS) and two previously reported polyketide synthase (PKS) genes (Castell-Miller et al., 2016) were DEGs; the NPS (DN5204_c0_g1, 3163_g) was similar to those in the expanded NPS-like (NPS1/NPS3/NPS13) group associated to phytotoxin production in *Cochliobolus* spp. (Condon et al., 2013) and one of the PKS matched the PKS7 of *Cochliobolus heterostrophus* (AAR90262; DN46786, 2466_g). Several putative fungal peptidases associated with pathogenesis were a metallo- (e.g., DN63292_c0_g1, 9982_g), a carboxy- (e.g., DN82009_c0_g1, 6849_g), a di- (DN14217_c0_g1, 2501_g), and a tripeptidyl peptidase (DN22434_c0_g1, 3704_g), as well as an extracellular metalloproteinase (DN11374_c0_g1, 3704_g) (**Supplementary Table 14**). Additional DEGs associated with pathogenicity and virulence included carbohydrate active enzymes (CAZymes), monooxygenases, regulatory proteins, an MFS antiporter protein, and toxins/mycotoxins (**Supplementary Table 14**).

### 3.5 RT-qPCR gene validation

The expression of six NWR and five fungal genes associated with defense and virulence, respectively, was validated by RT-qPCR. Overall, gene expression results agreed with those observed in the RNA sequencing study (**Supplementary Figures 6**, **7**). However, higher expression was found for the ent-cassadiene C11-alpha-hydroxylase (hereafter C7M76) and subtilisin-chymotrypsin inhibitor (hereafter ICI) by the RT-qPCR assay at 48 h than in the RNA sequencing study. Discrepancies like this can occur because the RT-qPCR is a more specific and precise method to assess gene expression than the use of whole genome transcriptome analysis. NWR gene expression was statistically significantly different between time points in RT-qPCR for the putative LRR receptor kinase SERK2 (ZPchr0011g27000), receptor-like protein EIX2 (ZPchr0002g25015), ent-cassadiene C11-alpha-hydroxylase C7M76 (ZPchr0006g43762), and a subtilisin-chymotrypsin inhibitor ICI (ZPchr0010g8932), but not for the chitin elicitor receptor kinase CERK1 (ZPchr0009g1619) or the respiratory burst oxidase homolog protein RBOHB (ZPchr0011g27322) (**Supplementary Figure 6**). In NWR, IF4D was identified as the most stable RG (Π = 1.00) followed by the IF1D (Π = 1.68) and G3DP (Π = 3.00) genes. Statistically differential temporal expression was observed for the *B. oryzae* NPS (3163_g), and the secreted isochorismatase effector (Isc1; 2994_g), but not for the reducing PKS (2466_g), isocytrate lyase 1 (4985_g), or the carboxypeptidase (6849_g) genes tested (**Supplementary Figure 7**). In *B. oryzae*, MitDiv1 was identified as the most stable RG (Π = 1.00), followed by Ubiq (Π = 1.68) and the G3DP (Π = 3.00) genes.

## 4 Discussion

*Bipolaris oryzae* is one of the most consequential threats to the NWR grain production and germplasm continuity in natural and cultivated environments (Johnson and Percich, 1992; Samac and Castell-Miller, 2023). To expand our understanding of plant defense and fungal virulence mechanisms, we designed an RNA sequencing study using an improved disease-resistant cultivar, Itasca-C12 (Porter, 2008), and a virulent *B. oryzae* isolate TG12Lb2 (Castell-Miller et al., 2016), during the first 48 h of their interaction.

We assembled and annotated the non-redundant reference transcriptome of NWR in the absence and in the presence of the fungus at 24 h and 48 h after inoculation (t_WRim) and that of *B. oryzae* with genes expressed *in vitro* and *in planta* at the same time points (t_Boiv_ip). The t_WRim resulted in a total of 93,610 transcripts (N50 = 1,656 bp), which roughly represents 13.6% of those initially generated in the NWR whole genome transcriptome (WGT) (Haas et al., 2021). The larger length (over 172 bp) of N50 transcripts in this study could be attributed to a reduced subset of expressed genes from mock- and *B. oryzae*-infected leaves compared with those of the WGT that was based on mostly all plant organ tissues (Haas et al., 2021). The percentage of NWR transcript annotations in this study that matched reference NWR genes (~63%) was similar to that found during the genome annotation project (Haas et al., 2021). The current version of the draft NWR genome has some issues with resolution of a high percentage of repetitive sequences (76.4%). Additional challenges in our NWR transcript annotations could be attributed to the heterogeneous and heterozygous nature of NWR, gene duplication, and expression of numerous alternative splicing variants that were not completely collapsed by CD-HIT program (Haas et al., 2021). In this study, we selected relatively strict thresholds for BLAST searches to reduce “false positive” matches for assigning functions, which could have some effect on results of transcripts with no annotation (“false negatives”). This could also affect the number of NWR transcripts matching genes in other databases. The t_Boiv_ip contained 20,385 transcripts (N50 = 1,767 bp). All annotated DEGs at 48 h in this study were found in the total 10,674 fungal transcripts from infected NWR used to support the fungal genome assembly (Castell-Miller et al., 2016), and 94% matched a *B. oryzae* isolate from white rice (Condon et al., 2013). Our analyses were based on *de novo* transcriptome assemblies, and thus, the differential expression performed on transcripts of this pathosystem was independent of the draft genomes and had no effect on the evaluation and interpretation of downstream analyses.

Fungal infection triggered a distinct response of plant gene expression over time (**Figures 2**, **3**; **Table 2**) compared to mock inoculation. The heatmap displayed distinct expression patterns in over half of the genes in response to fungal infection (upregulation) compared to mock inoculation (downregulation). This suggests a coordinated and specific response to the pathogen. Despite the less distinct expression of these genes at different time points, there was a greater stability at 48 h for both infected and mock groups. This could indicate a longer-lasting change in gene expression patterns for adaptation to environmental and/or biotic stress. In line with this, at least two principal components (PC1 = 32.2% and PC2 = 14.5%), which explained the variability in gene expression, were strongly associated with the treatments and collection time, respectively (**Supplementary Figure 4**).

The number of NWR DEGs between fungal-infected and fungal-free plants increased by more than 2.6-fold over time (**Table 2**). Although there were no significant GO enrichments in this set, many of the upregulated DEGs were associated with defense against biotic threats. The heightened activation of specific NWR genes in reaction to the existence of the fungus holds biological significance, as they possess functional annotations related to protection against biotic stresses (**Supplementary Table 13**). These genes included membrane and cytoplasmic-localized receptors, downstream signaling events, elicitation of transcription factors, and the synthesis of various defense proteins, phytohormones, and antimicrobial secondary metabolites that are known to play a role in defense against pathogens (Wu et al., 2011; De Vleesschauwer et al., 2013; Camejo et al., 2016; Boutrot and Zipfel, 2017; Aldon et al., 2018; Chen et al., 2021). These events occur within a short time frame of 48 h, and their exact sequence and timing are not fully understood. However, this response is crucial for the plant’s defense against the fungus and highlights the complex and rapid nature of the plant’s immune system. This study has resulted in the initial framework for unraveling the interaction between NWR and *B. oryzae* (**Supplementary Figure 8**).

Heatmaps showed visual contrasts between the transcriptional profiles of the fungus grown *in vitro* and *in planta*, highlighting its unique strategies for survival and growth in these different environments (**Figure 2**). Time had less impact on gene expression, except for fungal growth *in planta*, where gene expression was more stable at 48 h and could indicate opposition to NWR gene expression. In agreement, the PCA detected that the conditions in which the fungus grows played a significant role in the variation of gene expression, with at least one PC with a strong association with treatments (PC1 = 28.4%) and another with a strong association with collection time (PC2 = 25%). Fungal DEG number increased over 2.3-fold from 24 h to 48 h. GO enrichments depicted that association with the host increased from 24 h to 48 h indicating an escalation in the plant–pathogen interaction. This likely is indicative of an extraordinary transcriptional and translational reprogramming involved not only in ordinary fungal metabolism for nutrient assimilation for growth and survival, but also for plant cell wall degradation (**Supplementary Table 8**). Additionally, some of the upregulated fungal genes, including effectors, were important factors in the variancePartition analysis (**Figure 4**). The data indicated fungal reprogramming for growth and pathogenesis over time as the engagement with NWR cellular defense activation progressed.

Plants have developed complex innate immune defenses against pathogens, which try to overcome plant tissue barriers, and/or suppress or manipulate their defenses (Toruño et al., 2016; Han and Kahmann, 2019; Shao et al., 2021). *B. oryzae* is generally considered a necrotroph (Condon et al., 2013), but it has also been suggested to have a short biotrophic stage (De Vleesschauwer et al., 2013; Thuy et al., 2023).

A major trigger for plant defenses is the detection of fungal chitin, the main fungal cell wall component, and its oligosaccharides (Vaghela et al., 2022). NWR DEGs with putative homology to two cell-surface LysM receptors, the RLP CEBiP (Kaku et al., 2006), upregulated at both time points, and the RLK OsCERK1 (Shimizu et al., 2010), upregulated at 48 h, were found. The RLP is essential for perception and binding to chitin derivatives through the external central LysM1 domain inducing dimerization (Hayafune et al., 2014), while OsCERK1 is required for chitin-elicitor signaling after hetero-oligomerization with CEBiP (Shimizu et al., 2010). In rice, chitin perception starts a series of downstream activation events (Akamatsu et al., 2013) including production of ROS, diterpenoid phytoalexins, callose deposition, and expression of basal defense genes (Shimizu et al., 2010; Ao et al., 2014). Both LysM chitin-induced receptors are needed to activate induced systemic resistance in rice against *B. oryzae* (Takagi et al., 2022). Perception of fungal chitin also activates acidic, exo-, and basic endo-chitinases (Vaghela et al., 2022). NWR produced transcripts similar to the rice vacuolar chitinases CHI1_ORYSJ (DN5450_c0_g1) and CHI2_ORYSJ (DN83338_c0_g1) that could have comparative defense roles as in rice (Datta et al., 2001). Within the most upregulated putative NWR TFs that could be linked to defense against fungi and response to chitin was a putative basic helix–loop–helix (*bHLH041*) (DN25752_c0_g1) gene. In wheat, a similar gene was uncovered as a participant candidate in a QTL conferring resistance to Fusarium head blight (Dhokane et al., 2016). *B. oryzae* also differentially secreted a protein with LysM domains (PF01476.21) and signal peptide (DN72040_c0_g1; CM_2799_g; Castell-Miller et al., 2016) matching *Bipolaris/Cochliobolus* spp. genes of carbohydrate binding module family 50, able to bind chitin and derivatives. It also matches, albeit with low similarity, the intracellular hyphae protein 1 of *Colletotrichum lindemuthianum*, important for its interaction with bean, including maintenance of its biotrophic phase (Perfect et al., 2000), and to the virulence effector *Ecp*6, a chitin-binding lectin (Bolton et al., 2008) from *C fulvum*; the latter avoids elicitation of PTI defenses (de Jonge et al., 2010) by outcompeting with AtCERK1 ectodomain receptor binding (Sánchez-Vallet et al., 2013), thus contributing to virulence.

Fungal endo-1,4-β-xylanases and a putative acetylxylan esterase, capable of hydrolysis of plant cell wall xylan, were differently expressed. In *Botrytis cinerea*, some xylanases also act as effectors inducing HR-like, ROS production and electrolyte leakage (Shao et al., 2021). Recently, a *B. oryzae* xylanase was reported as an apoplastic effector eliciting defenses in a Korean rice variety (Lee et al., 2023). NWR highly expresses xylanase inhibitors like those from rice (e.g., XIP-1 gene), which have competitive inhibitory activity toward enzymatic degradative activity of fungal xylanases (Durand et al., 2005). Other *B. oryzae* DEGs were two apoplastic effectors, pectate lyases, and a rhamnogalacturonan acetylesterase able to degrade pectin, present in the extracellular matrix of plant cell walls releasing oligogalacturonides (De Lorenzo et al., 2011). Putative NWR WAK membrane-bound receptors, capable of binding pectin and derivatives, were also DEGs. WAKs can activate MAPKs and other signaling pathways in response to cell wall disturbance (Kohorn, 2015). WAK receptors are involved in resistance to the spot blotch pathogen *B. sorokiniana* in barley at the seedling stage (Ameen et al., 2020) and to the hemibiotrophic *Exserohilum turcicum* causing northern corn leaf blight (Yang et al., 2019). Transcription of WAK genes through chitin detection in association with the CEBiP receptor was reported (Delteil et al., 2016). *B. oryzae* peptidases were differentially expressed. They could have a potential role as effectors and facilitate host colonization (Franceschetti et al., 2017). An extracellular metalloproteinase also carried a fungalysin domain, which is present in some effectors (Shao et al., 2021) like the gene Cgfl from *Collectotrichum graminicola* that is highly transcribed during the conversion from the biotrophic to the necrotrophic phase of corn colonization (Vargas et al., 2012), and could cleave plant chitinases (Sanz-Martín et al., 2016). A few NWR cysteine and serine protease inhibitors were also activated. Many other *B. oryzae* cell wall-degrading enzymes were also DEGs and found in NWR and white rice studies (Castell-Miller et al., 2016; Lee et al., 2023; Singh et al., 2023).

Among the most upregulated DEG matching receptors were several associated with plant defenses, such as SERK2, RLPK Feronia, and EIX2. SERK2 interprets cues of complex signaling networks from other surface-localized proteins and precisely modulates cellular responses including MAPK, Ca^2+^, ROS, and defense gene expression including phytoalexin production (Ma et al., 2016). It is associated with quantitative disease resistance to *Sclerotinia sclerotiorum* in Arabidopsis (Derbyshire et al., 2019) and positively regulates defenses against the blast fungus *Magnaporthe oryzae* in rice (Hu et al., 2005). The RLPK Feronia is another multi-functional receptor monitoring cell wall integrity (Kohorn, 2015) and defenses against *M. oryzae* in response to Ca^2+^ deficiency (Luo et al., 2022), but in tomato, it could facilitate fungal pathogenicity through mediating the Rapid Alkalinization Factor (RAFL)-FER pathway (Masachis et al., 2016). Both receptors in our pathosystem had a strong effect on treatment in the variancePartition analysis. EIX2 receptor (DN12795_c0_g1) is involved in receptor-mediated endocytosis and induction of HR (Ron and Avni, 2004).

Upon perception of fungal-derived conserved molecules, plant cell ROS homeostasis is altered (Camejo et al., 2016; Kawasaki et al., 2017). RBHOs are calcium-dependent NADPH oxidases that generate superoxide (Camejo et al., 2016) and participate in plant defenses. In our study, three potential NWR RBOH genes were DEGs, including an RBOHB and two RBHOA, of which ZPchr0010g9242 has a Ca^2+^ binding domain that could be involved in regulation of proteins (Torres and Dangl, 2005). Other DEGs were several peroxidases that can participate in the fortification of cell walls, detoxification (Camejo et al., 2016), and regulation of hormone signaling pathways of other PRPs and phytoalexins (Almagro et al., 2009). Involvement of ROS in rice defenses against *B. oryzae* has been reported (Ashfaq et al., 2021; Thuy et al., 2023). In our study, some of these genes were found within plant–pathogen interaction and MAPK KEGG biological pathways, and top drivers in the variancePartition due to treatments (**Supplementary Table 13**), suggesting a strong ROS response during *B. oryzae* attack. The association of the complex signaling networking of RBOH genes with Ca^2+^, CDPK, and protein phosphorylation has been established in crops under abiotic and biotic (e.g., PAMPs) stresses (Torres and Dangl, 2005; Camejo et al., 2016).

Many NWR DEGs were associated with Ca^2+^ signaling pathways including plasma membrane receptors/ion channels involved in Ca^2+^ movement into the cell (Park and Shin, 2022), and Ca^2+^ binding sensors can detect spatiotemporal changes in the Ca^2+^ cytosolic content and, in turn, activate TFs, kinases, ROS, PRPs, and phytohormones (Cheval et al., 2013; Zhang et al., 2014; Aldon et al., 2018). Other associated genes included a putative NWR transcription regulator belonging to the CBP60 family, conserved in defenses against pathogens (Kumari et al., 2022), as well as an MLO-like protein, linked to the modulation of pathogen defenses (broad-spectrum) and programmed cell death in rice leaves (Kim et al., 2002). Ca^2+^ signaling genes were part of the plant–pathogen interaction and plant MAPK signaling KEGG enriched pathways (WGCNA), and a driving force in the variancePartition analysis.

Other signal molecules, MAPKs, were also differently expressed in our RNA sequencing study. Some of these MAPKs (M3K17 and M2K5) could be involved in abscisic acid regulation (Li et al., 2017), especially M2K5 that participates in ABA rice defense against *B. oryzae* (De Vleesschauwer et al., 2010). Another NWR transcript (DN4590_c0_g1) had high similarity to MPK12_ORYSJ (also called BWMK1), a gene activated by diverse stimuli including the rice pathogen *M. oryzae*, wounding, and ROS (He et al., 1999; Cheong et al., 2003) and in turn phosphorylates a TF that could interact with several PRPs and play a part in resistance to pathogens (Cheong et al., 2003).

Phytohormones involved in signaling processes have complex interactive networking that allows elicitation of specific defenses (Checker et al., 2018). In Arabidopsis, the SA signaling pathway mostly induces defenses against biotrophic stages of pathogens (Glazebrook, 2005) while the balancing action between/among JA, ET, and ABA pathways appears activated against some necrotrophic pathogens (Glazebrook, 2005; De Vleesschauwer et al., 2014; Ghozlan et al., 2020). However, rice phytohormone signaling does not appear to support the binary Arabidopsis model or to be tightly linked to the pathogen lifestyle, and it can even have participation of non-canonical phytohormones (De Vleesschauwer et al., 2013).

SA is an important component of plant immunity (Glazebrook, 2005; Qi et al., 2018) involved in local and systemic defense responses. SA signaling is linked to activation of defense-responsive genes (De Vleesschauwer et al., 2014) like PRPs and secondary metabolites (Glazebrook, 2005). In rice and in *Brachypodium distachyon*, sodium salicylate applications contributed to resistance against *Rhizoctonia solani* (Kouzai et al., 2018), and plants deficient in the production of SA (NahG mutants) were more susceptible. *R. solani* is considered a necrotroph; thus, it was suggested that a short biotrophic stage could be the target of rice SA defenses (Kouzai et al., 2018). NWR SA production can also be an attempt to control a possible subtle biotrophic phase of *B. oryzae* (De Vleesschauwer et al., 2013; Thuy et al., 2023). In our study, a putative *B. oryzae* salicylate hydroxylase enzyme (e.g., 7642_g) was highly upregulated, and it could participate in the degradation of aromatic compounds like SA or derivatives (Qi et al., 2018), or might alter the plant redox homeostasis provided by SA and thus promote oxidative damage (Yang et al., 2004) to favor its necrotrophic lifestyle phase. Moreover, *B. oryzae* also expresses a putative secreted ISC1 gene (DN46467_c0_g1; 4985_g), similar to the unorthodox effector from *V. dahliae*, which, in cotton, is required for complete pathogenicity by manipulating the SA metabolic pathways and suppressing defenses (Liu et al., 2014). However, the effectiveness of rice SA against *B. oryzae* infection was downplayed in other studies (Songkumarn et al., 2019).

JA and derivatives have a central role in rice defense signaling against pathogens with diverse lifestyles (De Vleesschauwer et al., 2013) with the majority of studies supporting jasmonates as positive regulators of disease control caused by hemibiotrophic (De Vleesschauwer et al., 2014) and some predominantly necrotrophic fungi (Kumar et al., 2023). However, in rice, JA-related genes were not effective defenses against *B. oryzae* (Ahn et al., 2005; De Vleesschauwer et al., 2014), or they have similar expression patterns in resistant and susceptible rice (Songkumarn et al., 2019). Other potential suggested roles support amplification of defense genes in response to diverse stressors (De Vleesschauwer et al., 2013), interacting with specific TFs to redirect the assistance of systemic defenses toward pathogen-inflicted wounds (Lorenzo et al., 2003), and regulation of ROS homeostasis in rice to alleviate oxidative ROS damage (Kumar et al., 2023).

ET immunity signaling appears to be complex and pathogen-dependent (De Vleesschauwer et al., 2014). In rice, production of ET positively restricts *M. oryzae* and *R. solani* invasion (De Vleesschauwer et al., 2013), but it acts as a strong suppressor of defenses against *B. oryzae* especially in susceptible cultivars (De Vleesschauwer et al., 2010). Several NWR ET-responsive transcription factors (ERF) were upregulated DEGs that could activate PRPs (Abiri et al., 2017) and participate in the regulation of ethylene/jasmonate pathways (Lorenzo et al., 2003) and, thus, warrant further investigation in this pathosystem.

The ABA signaling pathway has been found to be important for defense against *B. oryzae* in rice (De Vleesschauwer et al., 2010). External ABA application on leaves restricted fungal colonization in mesophyll cells by negating signaling expression of plant ET, which is thought to be induced by the fungus and promotes susceptibility. The ABA signaling occurs in an OsMPK5-dependent manner and with the recruitment of a heterotrimeric alpha subunit of a G-protein (De Vleesschauwer et al., 2010). This outcome is independent of SA and JA signaling pathways or callose deposition. ABA also appears to tune the effect of ROS dispersal and quantity over time on epidermal and mesophyll rice cells upon *B. oryzae* infection (De Vleesschauwer et al., 2010). Some NWR DEGs associated with ABA biosynthesis and the ABA signaling pathway were activated in reaction to *B. oryzae* infection (**Supplementary Table 13**). For example, an NWR transcript in connection with ABA defenses was the exocytosis pathway E70B1, a positive regulator of ABA that assists in resistance against *M. oryzae* through perception of chitin and potentially interaction with CERK1 (Hou et al., 2020). Other ABA-related DEGs were MAPKs and GTPases.

Further research is needed to fully understand the specific roles of DEGs annotated to phytohormones within this pathosystem.

Phytoalexins appear to play a major role in the *B. oryzae*–NWR interaction. NWR DEGs matched those for phytoalexin production in rice and *Z. latifolia*. Rice produces several antimicrobial compounds, most of which are labdane-related diterpenoids (Lu et al., 2018) including momilactones, oryzalexins, and phytocassanes (Piasecka et al., 2015; Schmelz et al., 2014; Valletta et al., 2023). Rice phytocassanes could play a role against pathogens such as *Magnaporthe oryzae* and *Xanthomonas oryzae* (Lu et al., 2018). The NWR PBGC contains mainly genes putatively involved in phytocassane synthesis with a possible gene duplication of a copalyl-diphosphate synthase, which appears unique for the NWR cluster. Within this PBGC was also a CYP76M5 that could aid in phytoalexin production (Wu et al., 2011) and a putative kaurene synthase of the syn-pathway, which, in rice, is involved in the generation of a precursor of oryzalexin S (Nemoto et al., 2004). Another NWR DEG had similarity to a rice momilactone A synthase. Momilactones could act as phytoalexins, function in allelopathy (Xu et al., 2012), and play a part in non-host disease resistance (Lu et al., 2018).

Within fungal transcripts were effector peptides that could contribute to the onset and development of disease (Toruño et al., 2016; Shao et al., 2021). The vast majority corresponded to peptides without annotation. Others had similarities to known virulence genes such as isocytrate lyase, ICL1, of the hemibiotroph *Leptosphaeria maculans* associated with pathogenesis to canola (*Brassica napus*) (Idnurm and Howlett, 2002) and to a NPS and a PKS with roles in growth and development, reproduction, response to oxidative stress, and pathogenicity toward plants and microorganisms (Turgeon and Bushley, 2010; Condon et al., 2013).

Our NWR and *B. oryzae* global expression findings are in agreement with other results in the early stages of interactions between monocots and *Bipolaris* spp. such as *B. sorghicola*–*Sorghum bicolor* L (Yazawa et al., 2013), *B. zeicola*–corn (Liu et al., 2015), and recently published transcriptomes of the rice–*B. oryzae* pathosystem (Marwein et al., 2022; Lee et al., 2023; Singh et al., 2023).

The use of dual transcriptome analyses proved to be successful in identifying NWR genes responsible for defending against *B. oryzae*, as well as those that the fungus could utilize to promote disease. These genes displayed varying levels of differential expression at both 24 h and 48 h after inoculation.

These findings serve as a valuable foundation for guiding future research objectives. FBS affects a majority of NWR genotypes due to the scarcity of resistance. While this disease can be managed with fungicides, breeding for resistance poses significant challenges due to the species’ cross-pollinated nature, its aquatic habitat, and the likely quantitative inheritance of this trait. The few cultivars with improved resistance (e.g., Itasca-C12) struggle to maintain resistance in the field as the pathogen population increases throughout the season. However, the study findings provide candidate defense genes and associated pathways that can be explored at additional time points and for allelic diversity at the population levels, which could lead to the identification of potential markers across a wide range of transcripts/genes for crop improvement. Indeed, the identification of co-expressed defense and pathogenicity proteins/effectors is key to a comprehensive understanding of the NWR and *B. oryzae* pathosystem.

## 5 Conclusions

This study is the foundation for gene discovery toward a better understanding of NWR innate immunity against *B. oryzae* and molecular mechanisms used by the fungus to cause disease during the early colonization process. The study uncovered the induction of active and timely transcriptional reprogramming of membrane-bound and cytoplasmic receptors, signaling mechanisms, and activation of known disease-associated defense genes and antimicrobial phytoalexins. The candidate genes provide new avenues of research for exploring gene expression and allele sequencing analyses in FBS-resistant and -susceptible genotypes to sort out their function. They also could help develop markers for selection of NWR genotypes with improved resistance to FBS. In addition, *B. oryzae* virulence genes could be targeted for CRISPR/Cas9-based mutagenesis or RNA interference for an enhanced understanding of pathogenesis and host responses.

## Data availability statement

The data presented in the study are deposited in the Data Repository for the University of Minnesota (DRUM), accession link: https://doi.org/10.13020/9kja-aj88.

## Author contributions

CC-M: Conceptualization, Formal analysis, Funding acquisition, Investigation, Methodology, Project administration, Resources, Supervision, Validation, Visualization, Writing – original draft, Writing – review & editing. TJYK: Data curation, Formal analysis, Resources, Validation, Visualization, Writing – review & editing. AR: Writing – review & editing. DCS: Software, Formal analysis, Writing – review & editing. DAS: Conceptualization, Funding acquisition, Investigation, Methodology, Project administration, Resources, Supervision, Writing – review & editing. JAK: Funding acquisition, Project administration, Resources, Supervision, Writing – review & editing.
