# Supplementary figures and images for: Interactive transcriptome analyses of Northern Wild Rice (Zizania palustris L.) and Bipolaris oryzae show convoluted communications during the early stages of fungal brown spot development
Source: Front Plant Sci. 2024 Apr 26;15:1350281. doi: 10.3389/fpls.2024.1350281 (PMC11086184; doi:10.3389/fpls.2024.1350281)

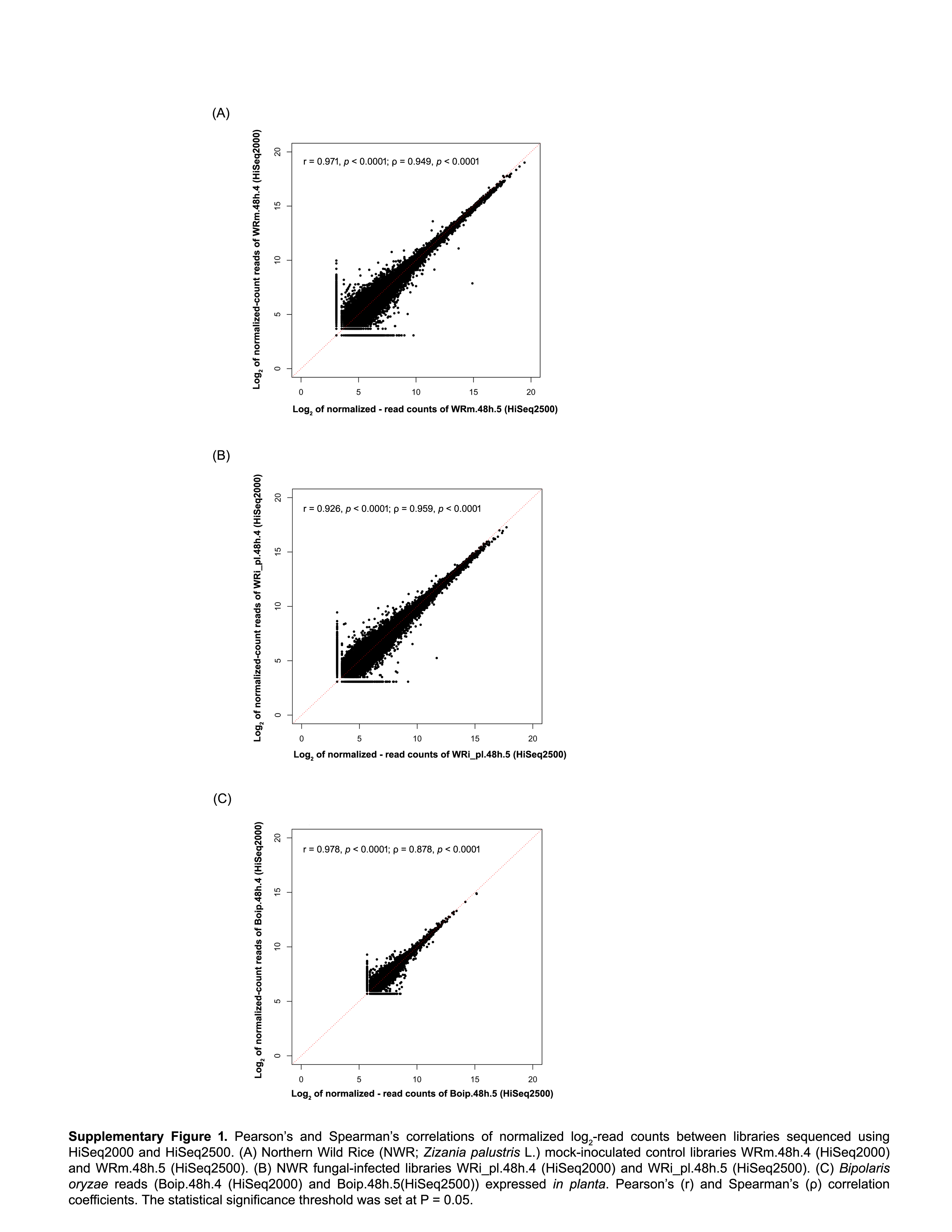

Supplement: Supplementary file 1 [file Image_1.tif]

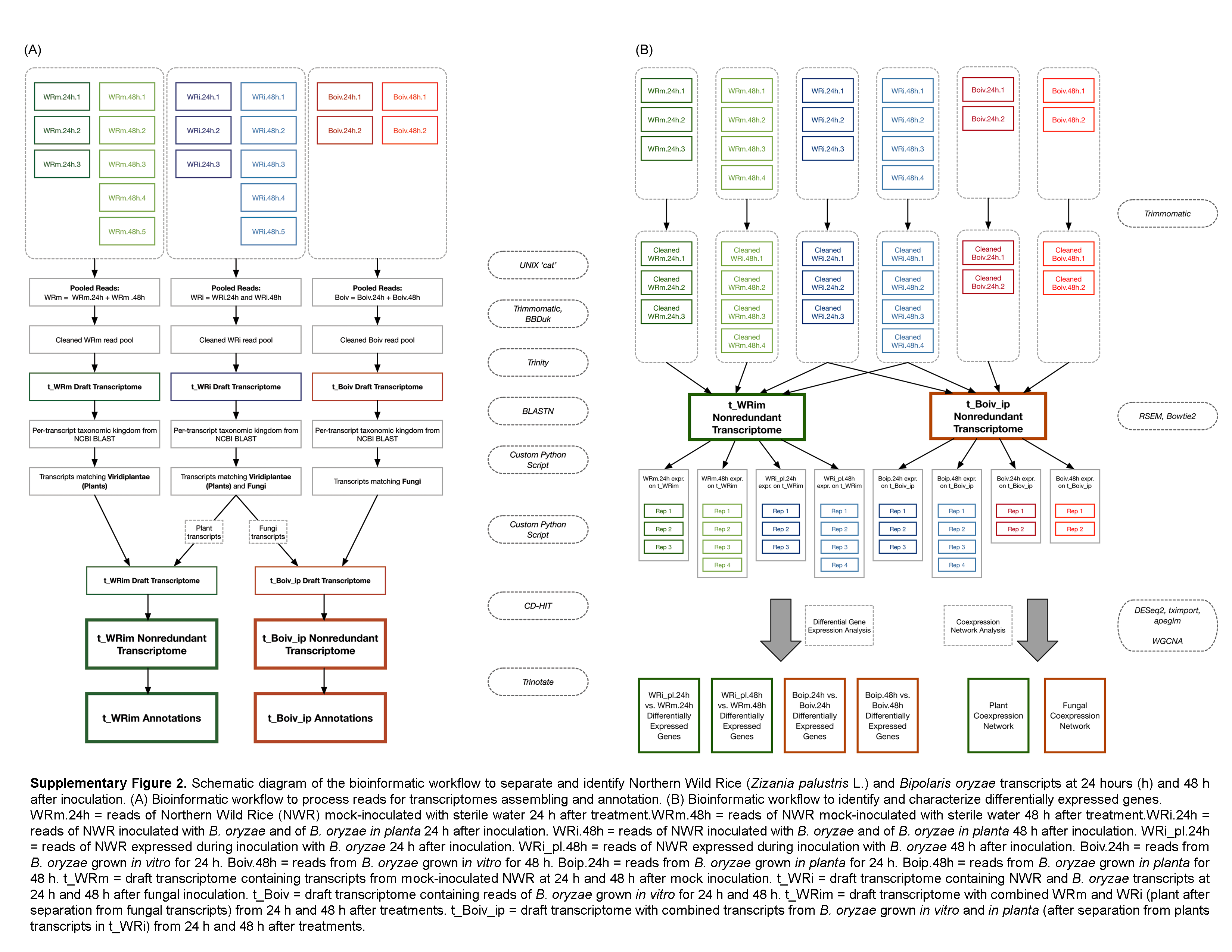

Supplement: Supplementary file 2 [file Image_2.tif]

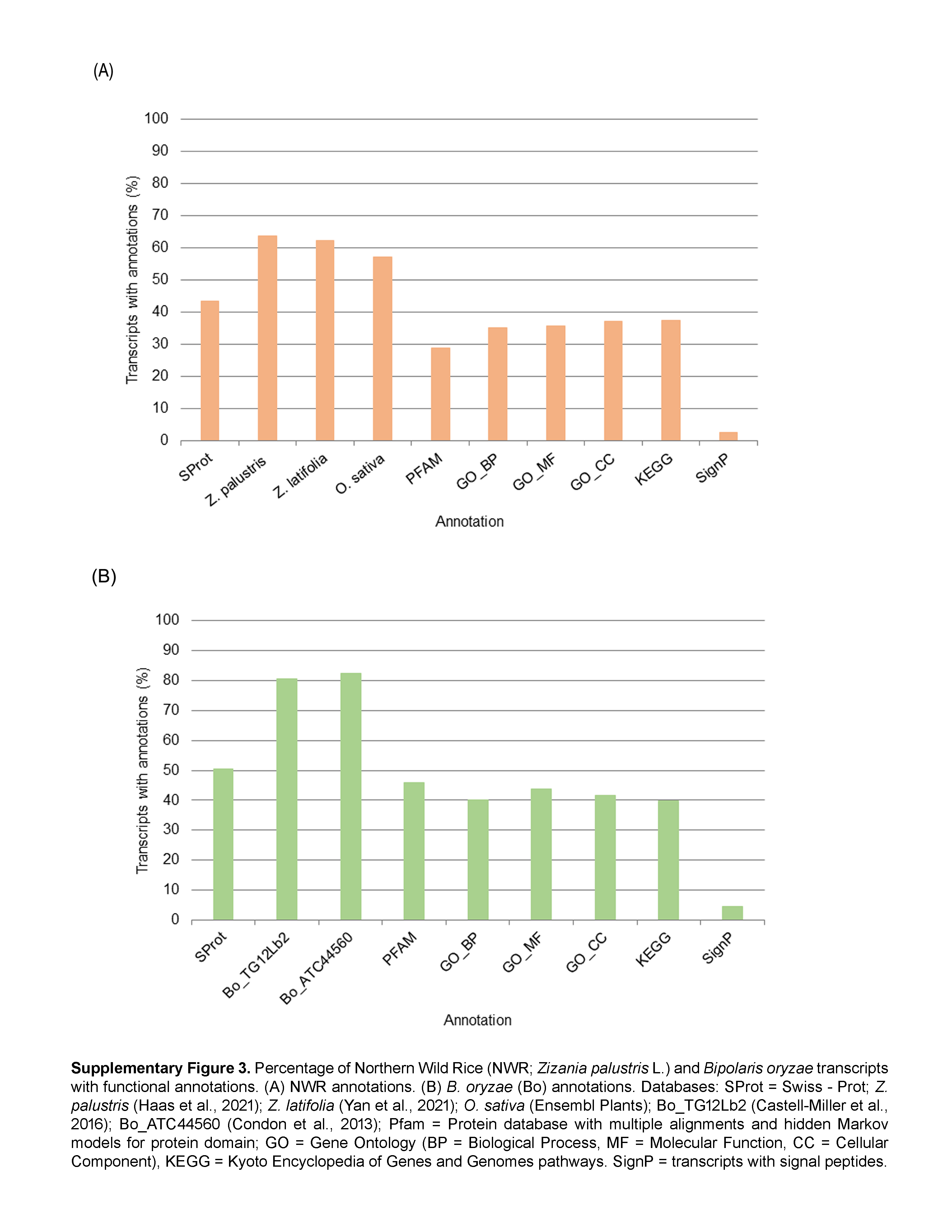

Supplement: Supplementary file 3 [file Image_3.tif]

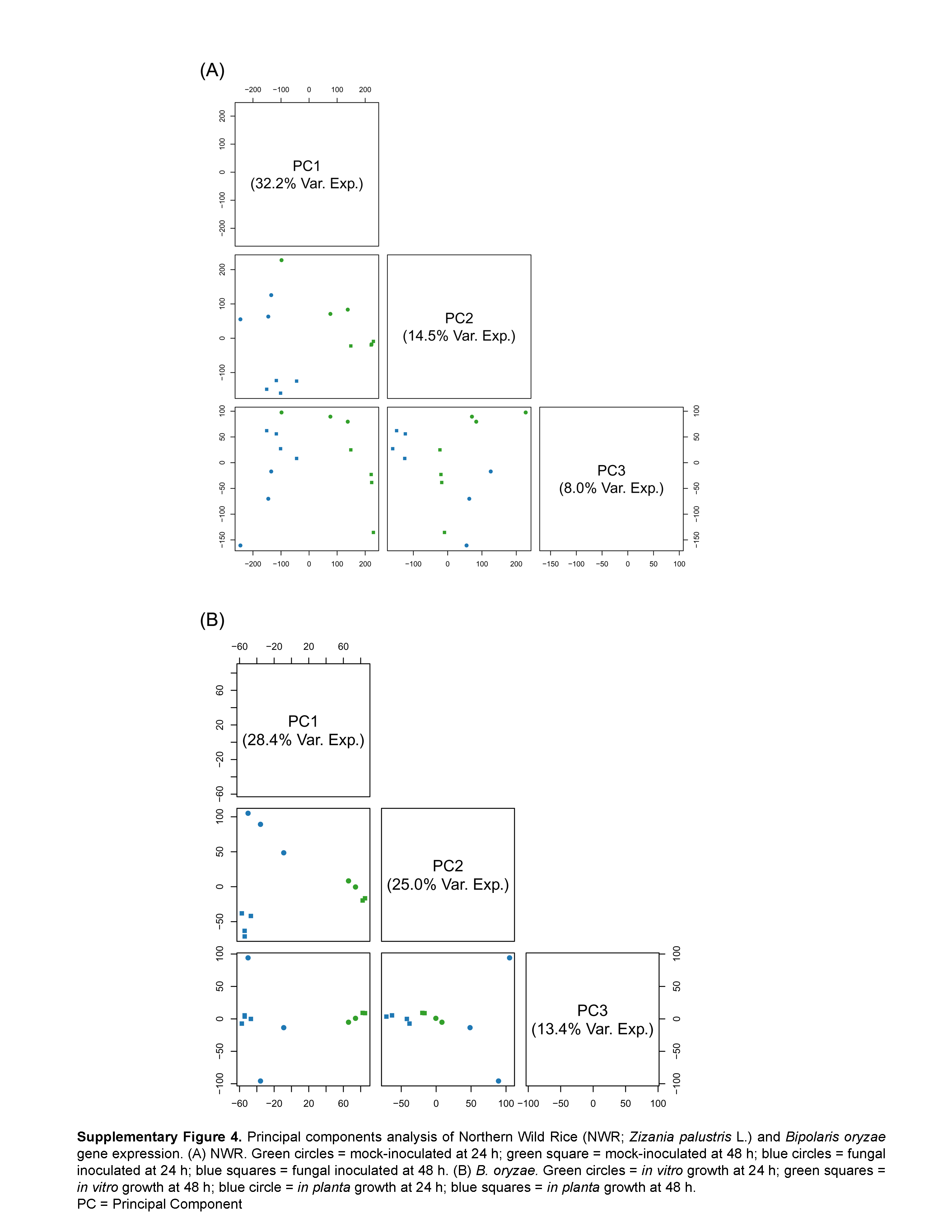

Supplement: Supplementary file 4 [file Image_4.tif]

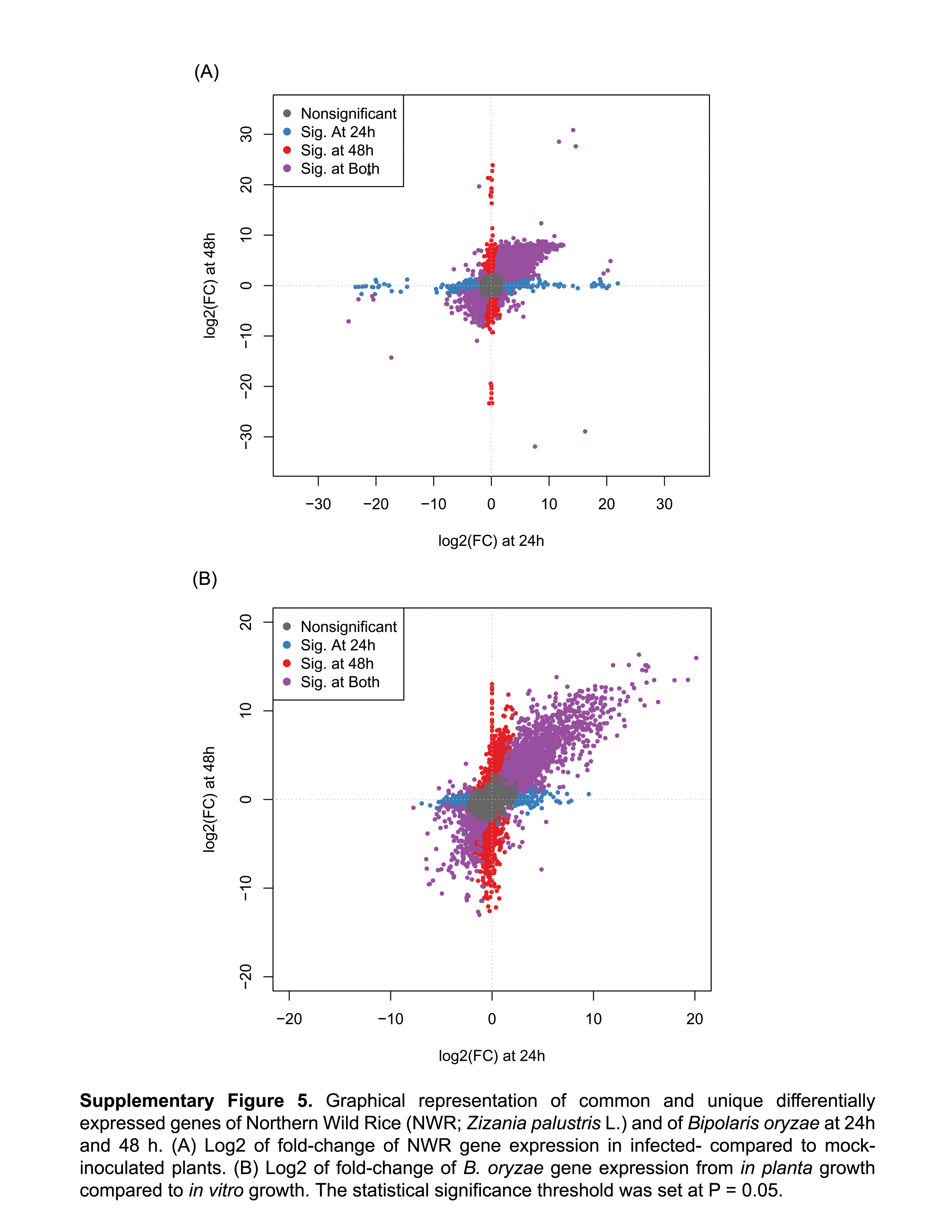

Supplement: Supplementary file 5 [file Image_5.tif]

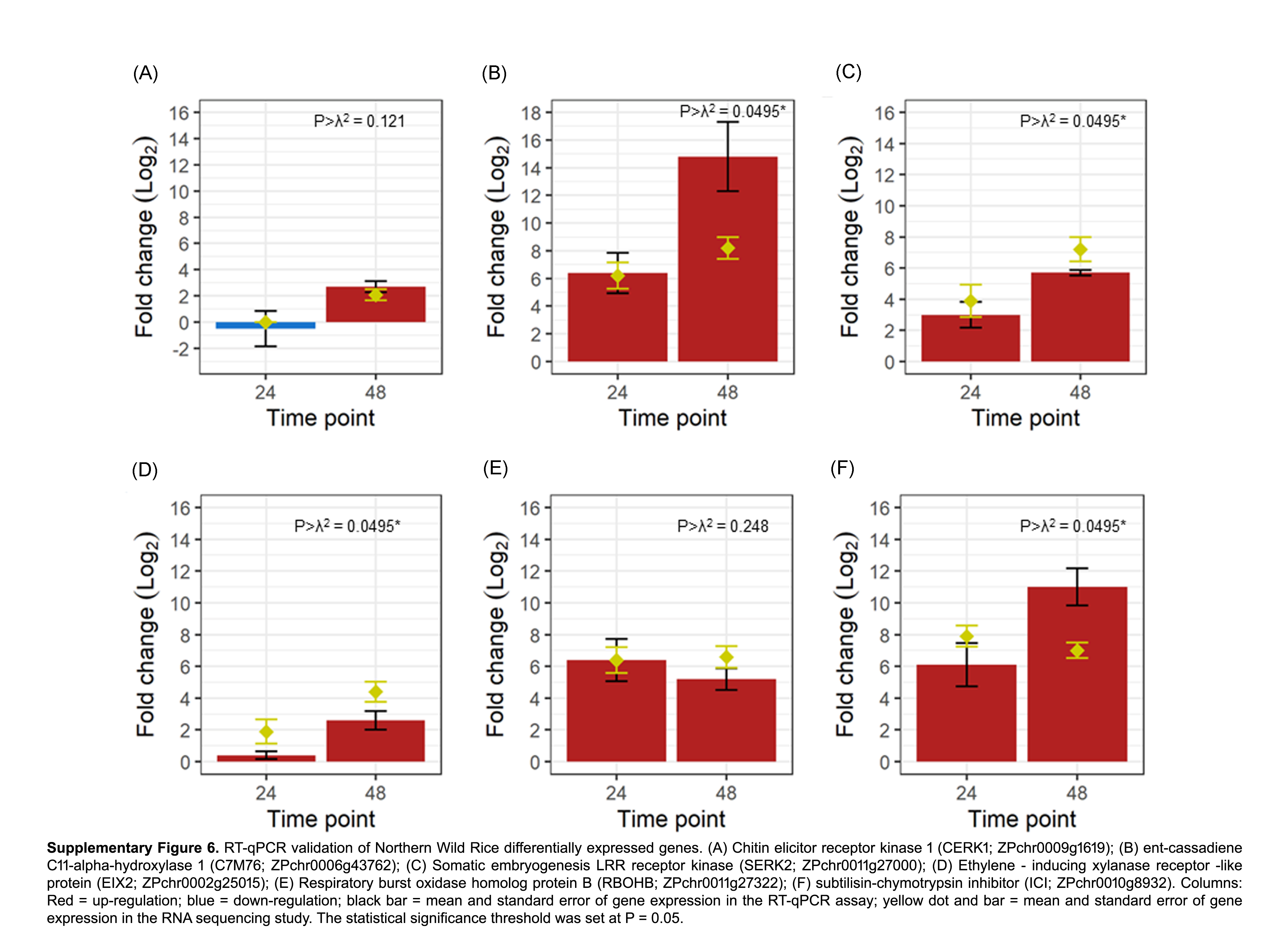

Supplement: Supplementary file 6 [file Image_6.tif]

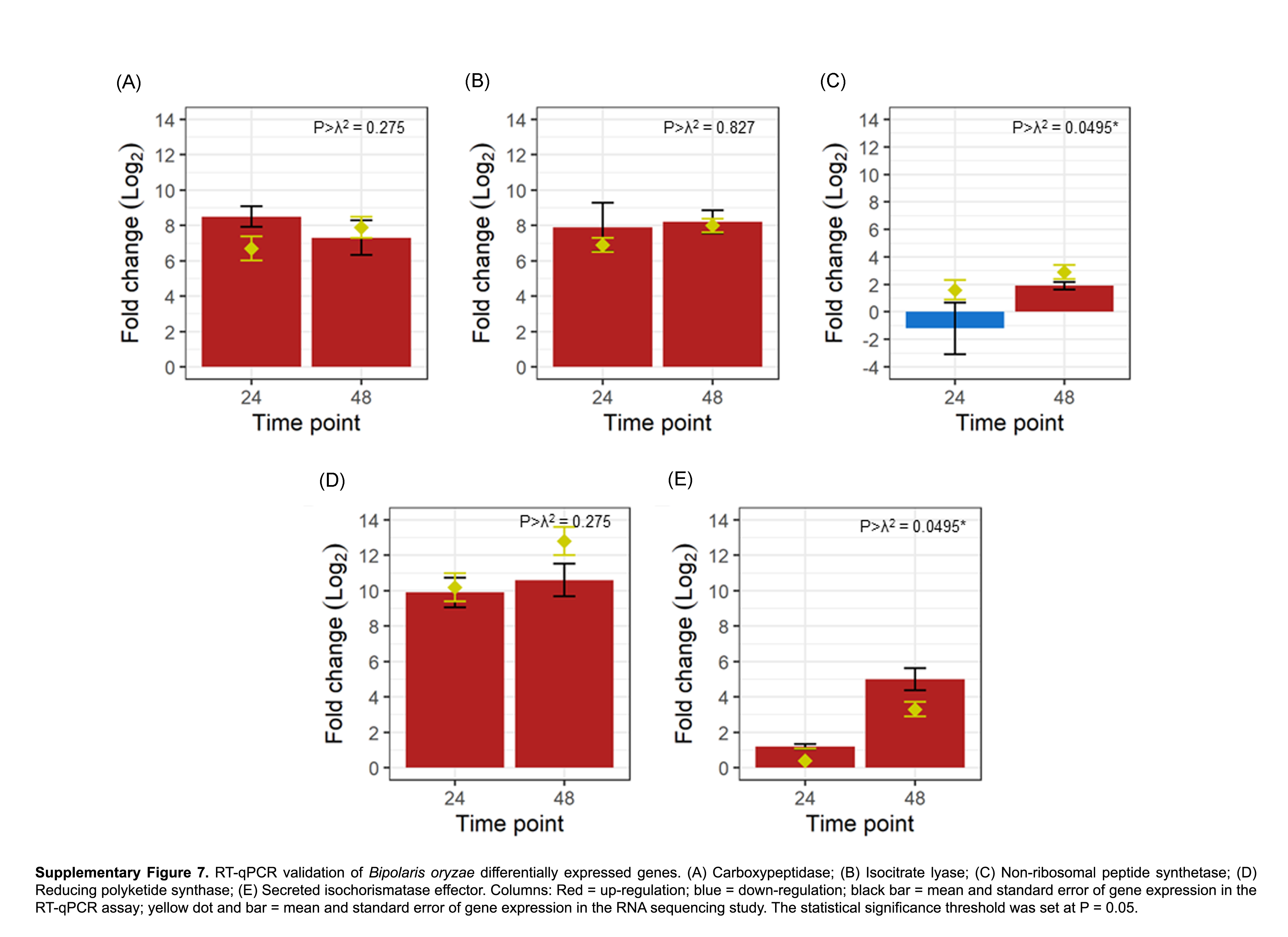

Supplement: Supplementary file 7 [file Image_7.tif]

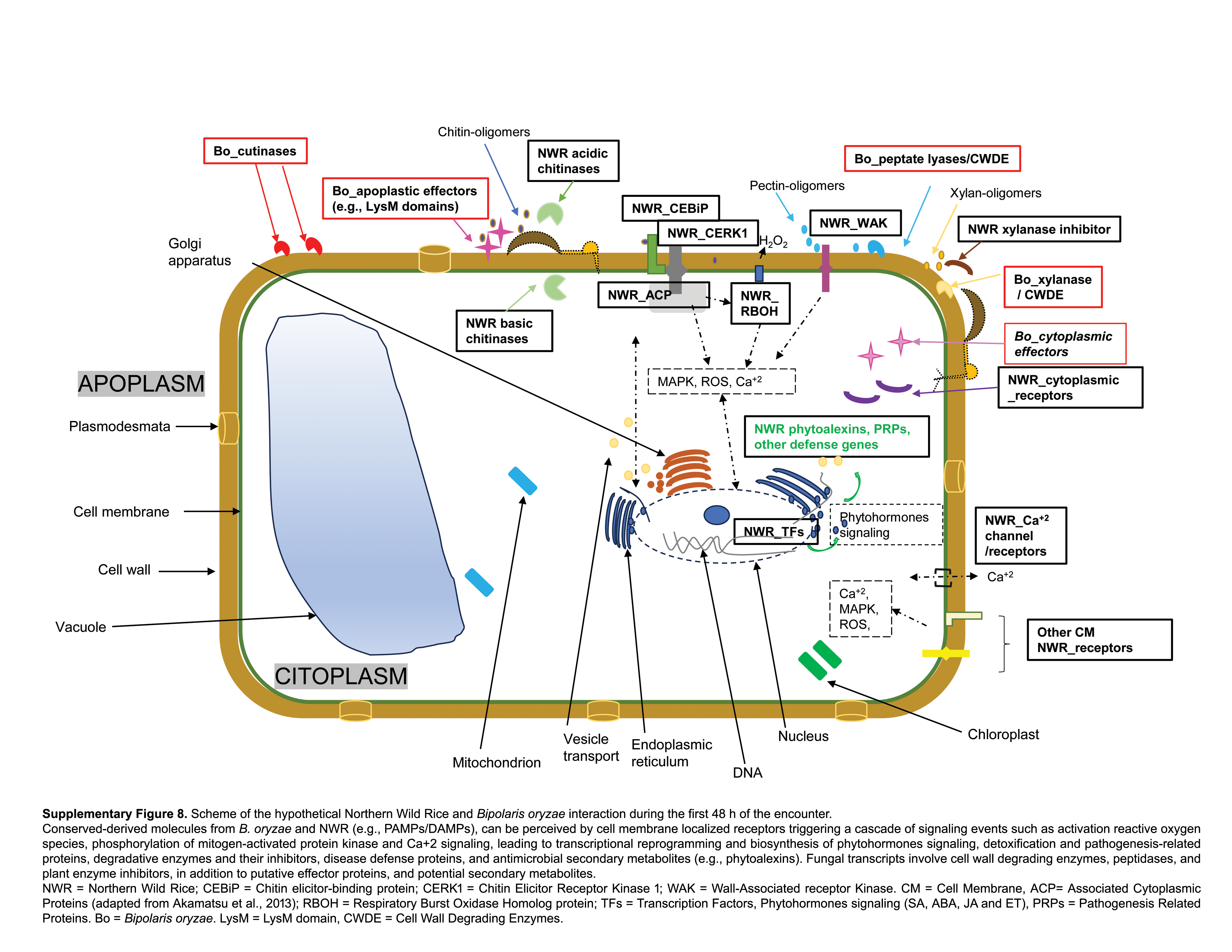

Supplement: Supplementary file 8 [file Image_8.tif]
