# Supplementary material for: Interactive transcriptome analyses of Northern Wild Rice (Zizania palustris L.) and Bipolaris oryzae show convoluted communications during the early stages of fungal brown spot development
Source: Front Plant Sci. 2024 Apr 26;15:1350281. doi: 10.3389/fpls.2024.1350281 (PMC11086184; doi:10.3389/fpls.2024.1350281)
Supplement: Supplementary file 9 [file Table_1.docx]

| **Supplementary Table 1.** Number of raw and high-quality single pass 50 bp reads of RNA_seq libraries sequenced with Illumina HiSeq2000 and HiSeq2500 technologies | | |
| --- | --- | --- |
| Library | Raw Reads | QC-passing Reads |
| Boiv.24h.1 | 38,369,953 | 38,135,201 |
| Boiv.24h.2 | 39,138,783 | 38,934,345 |
| Boiv.48h.1 | 37,195,919 | 36,978,253 |
| Boiv.48h.2 | 38,723,853 | 38,461,974 |
| WRi.24h.1 | 37,935,111 | 37,791,526 |
| WRi.24h.2 | 36,727,475 | 36,252,906 |
| WRi.24h.3 | 38,366,160 | 38,145,894 |
| WRi.48h.1 | 42,291,104 | 41,993,521 |
| WRi.48h.2 | 47,808,446 | 47,473,584 |
| WRi.48h.3 | 45,784,268 | 45,454,918 |
| WRi.48h.4 | 53,541,909 | 53,158,175 |
| WRi.48h.5* | 29,867,720 | 29,661,780 |
| WRm.24h.1 | 38,234,080 | 38,032,579 |
| WRm.24h.2 | 42,852,575 | 40,818,232 |
| WRm.24h.3 | 37,121,456 | 36,898,106 |
| WRm.48h.1 | 40,553,109 | 40,235,851 |
| WRm.48h.2 | 38,957,313 | 38,566,189 |
| WRm.48h.3 | 49,342,857 | 48,927,326 |
| WRm.48h.4 | 36,690,539 | 36,383,557 |
| WRm.48h.5* | 35,032,318 | 34,787,113 |

Boiv*= Bipolaris oryzae in vitro* growth reads; WRi = Northern Wild Rice (NWR) and *B. oryzae* reads; WRm = NWR mock – inoculated reads; 24 h and 48 h = collection hours after inoculation. Numbers 1 to 4 = number of biological replicates; * = 5 is a technical replicate of biological replicate 4 for testing consistency between HiSeq2000 and HiSeq2500 technologies.
