# Supplementary material for: Interactive transcriptome analyses of Northern Wild Rice (Zizania palustris L.) and Bipolaris oryzae show convoluted communications during the early stages of fungal brown spot development
Source: Front Plant Sci. 2024 Apr 26;15:1350281. doi: 10.3389/fpls.2024.1350281 (PMC11086184; doi:10.3389/fpls.2024.1350281)
Supplement: Supplementary file 10 [file Table_2.docx]

**Supplementary Table 2.** Summary of total normalized log_2_ read counts in Northern Wild Rice mock, fungal infected; and *Bipolaris oryzae* grown *in vitro* assemblies

| Pool | Raw reads | After rRNA removal | After adapter and quality trimming |
| --- | --- | --- | --- |
| WRm | 318,784,247 | 311,326,775 | 307,218,873 |
| WRi | 332,322,193 | 328,521,696 | 326,146,913 |
| Boiv | 153,428,508 | 143,600,218 | 142,686,855 |

WRm = Northern Wild Rice (NWR) mock – inoculated reads (WRm.24h.1, WRm.24h.2, WRm.24h.3, WRm.48h.1, WRm.48h.2, WRm.48h.3, WRm.48h.4, and WRm.48h.5), WRi = NWR fungal-infected and *B. oryzae* growing *in planta* reads (WRi.24h.1, WRi.24h.2, WRi.24h.3, WRi.48h.1, WRi.48h.2, WRi.48h.3, WRi.48h.4, and WRi.48h.5), Boiv = *B. oryzae* grown *in vitro* reads (Boiv.24h.1, Boiv.24h.2, Boiv.48h.1 and Boiv.48h.2).
