# Supplementary material for: Interactive transcriptome analyses of Northern Wild Rice (Zizania palustris L.) and Bipolaris oryzae show convoluted communications during the early stages of fungal brown spot development
Source: Front Plant Sci. 2024 Apr 26;15:1350281. doi: 10.3389/fpls.2024.1350281 (PMC11086184; doi:10.3389/fpls.2024.1350281)
Supplement: Supplementary file 11 [file Table_3.docx]

| **Supplementary Table 3.** Summary statistics for the draft Northern Wild Rice mock, fungal infected; and *Bipolaris oryzae* grown *in vitro* draft transcriptome assemblies. | | | | | | | | |
| --- | --- | --- | --- | --- | --- | --- | --- | --- |
|  | Transcripts | | | | | | |  |
| Transcriptome | Number | Length (bp) | | | | | | N50 (bp) |
|  |  | Minimum | Median | Mean | Std. Dev | Maximum | Total Assembly |  |
| t_WRm | 86,876 | 251 | 489.0 | 901.93 | 959.19 | 15,192 | 78,356,201 | 1,520 |
| t_WRi | 97,258 | 251 | 478.0 | 866.76 | 918.03 | 20,537 | 84,298,903 | 1,425 |
| t_Boiv | 27,684 | 251 | 926.0 | 1,227.47 | 1,009.35 | 10,819 | 33,981,368 | 1,827 |

t_WRm = Northern Wild Rice (NWR) mock-inoculated draft transcriptome, t_WRi = NWR fungal infected and *B. oryzae* growing *in planta* draft transcriptome; t_Boiv = *B. oryzae* grown *in*-*vitro* draft transcriptome. Each transcriptome contains transcripts assembled from normalized log_2_ reads collected at 24 h and 48 h after treatments.
