# Supplementary material for: Interactive transcriptome analyses of Northern Wild Rice (Zizania palustris L.) and Bipolaris oryzae show convoluted communications during the early stages of fungal brown spot development
Source: Front Plant Sci. 2024 Apr 26;15:1350281. doi: 10.3389/fpls.2024.1350281 (PMC11086184; doi:10.3389/fpls.2024.1350281)
Supplement: Supplementary file 12 [file Table_4.docx]

| **Supplementary Table 4.** Percent of genes completeness (BUSCO) for Northern Wild Rice mock, fungal infected; and *Bipolaris oryzae* grown *in vitro* draft transcriptomes | | | | | |
| --- | --- | --- | --- | --- | --- |
| Assembly | Percent of Gene | | | | N^1^ |
|  | Complete | (Single copy; Duplicated) | Fragmented | Missing |  |
| t_WRm  t_WRi t_Boiv | 68.2  69.5  90.2 | (31.5; 36.7)  (29.5; 40.0)  (18.4; 71.8) | 14.9  13.4  4.2 | 16.9  17.1  5.6 | 3236  3236  1706 |

t_WRm = Northern Wild Rice (NWR) mock-inoculated draft transcriptome, t_WRi = NWR fungal infected and *B. oryzae* growing *in planta* draft transcriptome; t_Boiv = *B. oryzae* grown *in* *vitro* draft transcriptome. Each transcriptome contains transcripts assembled from normalized log_2_ reads collected at 24 h and 48 h after treatments.

^1^ N=Number of orthologues tested.
