# Supplementary material for: Interactive transcriptome analyses of Northern Wild Rice (Zizania palustris L.) and Bipolaris oryzae show convoluted communications during the early stages of fungal brown spot development
Source: Front Plant Sci. 2024 Apr 26;15:1350281. doi: 10.3389/fpls.2024.1350281 (PMC11086184; doi:10.3389/fpls.2024.1350281)
Supplement: Supplementary file 13 [file Table_5.docx]

| **Supplementary Table 5.** Summary of percent single pass reads represented in the transcripts of Northern Wild Rice mock, fungal infected; and *Bipolaris oryzae* grown *in vitro* draft transcriptome assemblies | | | | |
| --- | --- | --- | --- | --- |
|  | SP read alignments (%) | | | |
| Assembly | Absence | Single | Multiple | Total |
| t_WRm  t_WRi  t_Boiv | 9.93  8.21  9.47 | 12.30  12.23  8.29 | 77.77  79.57  82.25 | 90.07  91.79  90.53 |

t_WRm = Northern Wild Rice (NWR) mock-inoculated draft transcriptome, t_WRi = NWR fungal infected and *B. oryzae* growing *in planta* draft transcriptome; t_Boiv = *B. oryzae* grown *in*-*vitro* draft transcriptome. Each transcriptome contains transcripts assembled from normalized log_2_ reads collected at 24 h and 48 h after treatments.
