# Supplementary material for: Interactive transcriptome analyses of Northern Wild Rice (Zizania palustris L.) and Bipolaris oryzae show convoluted communications during the early stages of fungal brown spot development
Source: Front Plant Sci. 2024 Apr 26;15:1350281. doi: 10.3389/fpls.2024.1350281 (PMC11086184; doi:10.3389/fpls.2024.1350281)
Supplement: Supplementary file 14 [file Table_6.docx]

| **Supplementary Table 6.** List of Pleosporales fungal databases used for *Bipolaris oryzae* TG12Lb2 transcriptome annotation | | | |
| --- | --- | --- | --- |
| Name | Taxon ID | Accession | Assembly |
| *Alternaria alternata* str. SRC1lrK2f  *Ascochyta rabiei* str. ArDII  *Bipolaris maydis* ATCC 48331  *Bipolaris maydis* C5  *Bipolaris oryzae* ATCC 44560  *Bipolaris sorokiniana* ND90Pr  *Bipolaris victoriae* FI3  *Bipolaris zeicola* 26-R-13  *Corynespora cassiicola* Philippines  *Epicoccum nigrum* str. ICMP 19927  *Exserohilum turcica* Et28A  *Leptosphaeria maculans*  *Paraphaeosphaeria sporulosa* str. AP3s5-JAC2a  *Periconia macrospinosa* str. DSE2036  *Phaeosphaeria nodorum*  *Pyrenochaeta* sp. DS3sAY3a  *Pyrenophora teres*  *Pyrenophora tritici-repentis*  *Pyrenophora tritici-repentis*  *Pyrenophora tritici-repentis*  *Pyrenophora tritici-repentis*  *Pyrenophora tritici-repentis* Pt-1C-BFP  *Stagonospora* sp. SRC1lsM3a  *Stemphylium lycopersici* str. CIDEFI 216 | 5599  5454  665024  701091  930090  665912  930091  930089  1448308  105696  671987  985895  1460663  97972  321614  765867  861557  45151  45151  45151  45151  426418  765868  183478 | GCA_001642055.1  GCA_001630375.1  GCA_000354255.1  GCA_000338975.1  GCA_000523455.1  GCA_000338995.1  GCA_000527765.1  GCA_000523435.1  GCA_003016335.1  GCA_002116315.1  GCA_000359705.1  GCA_000230375.1  GCA_001642045.1  GCA_003073855.1  GCA_000146915.1  GCA_001644535.1  GCA_000166005.1  GCA_003171545.1  GCA_003231325.1  GCA_003231365.1  GCA_003231425.1  GCA_000149985.1  GCA_001644525.1  GCA_001191545.1 | Altal1  AscRab1.0  CocheC4_1  CocheC5_3  Cochliobolus_miyabeanus_v1.0  Cocsa1  Cochliobolus_victoriae_v1.0  Cochliobolus_carbonum_v1.0  Corynespora_cassiicola_v1.0  ASM211631v1  Setospaeria_trucica_Et28A_v1.0  ASM23037v1  Parsp1  Perma1  ASM14691v1  Pyrsp1  GCA_000166005.1  PtrARCrossB10  Ptr134  Ptr239  Ptr86-124  ASM14998v1  Stasp1  ASM119154v1 |
