# Supplementary material for: Interactive transcriptome analyses of Northern Wild Rice (Zizania palustris L.) and Bipolaris oryzae show convoluted communications during the early stages of fungal brown spot development
Source: Front Plant Sci. 2024 Apr 26;15:1350281. doi: 10.3389/fpls.2024.1350281 (PMC11086184; doi:10.3389/fpls.2024.1350281)
Supplement: Supplementary file 23 [file DataSheet_1.docx]

**Supplementary Data 1.** **Illumina platform data reproducibility.**

The reproducibility of relative expression in the mock-inoculated plant libraries between the Illumina technologies was tested with the log_2_ of normalized read counts of WRm.48h.4 (HiSeq2000) and WRm.48h.5 (HiSeq2500). The reproducibility of relative expression in the fungal-challenged plant libraries (WRi) was tested after separation of the transcripts expressed by the plant during fungal infection (hereafter, WRi_pl.48h.4 and WRi_pl.48h.5) and between fungal transcripts expressed *in* *planta* (Boip.48h.4 and Boip.48h.5). Pearson’s and Spearman’s correlations were tested under the null hypothesis of no correlation among relative expression of both Illumina technologies, that is H_0_: r (Pearson’s “r” coefficient) = 0 and H_0_: *ρ* (Spearman’s “rho” coefficient) = 0, respectively, versus the alternative hypothesis that correlations were nonzero. The statistically significant level was set to P = 0.05.

Pearson’s and Spearman’s correlation coefficients were each statistically significantly greater than zero between all pairwise comparisons, rejecting the null hypothesis of no correlation between each set (Supplementary Figure 1). Similar results were obtained when the analysis was performed without separation of fungal and plant reads that is, with WRi.48h.4 and WRi.48h.5 libraries (data not shown). These results on technical control libraries indicated strong linear relationship between reads counts using both technologies, and thus, highly comparable.
