# Supplementary material for: Interactive transcriptome analyses of Northern Wild Rice (Zizania palustris L.) and Bipolaris oryzae show convoluted communications during the early stages of fungal brown spot development
Source: Front Plant Sci. 2024 Apr 26;15:1350281. doi: 10.3389/fpls.2024.1350281 (PMC11086184; doi:10.3389/fpls.2024.1350281)
Supplement: Supplementary file 24 [file DataSheet_2.docx]

**Supplementary Data 2. Draft NWR and *B. oryzae* transcriptome assemblies.**

For draft transcriptome assemblies, the raw SP reads of 20 libraries (Supplementary Table 1) were combined into three files containing each: A) SP reads of NWR mock inoculated (WRm), B) joint SP reads of NWR and the fungus *in* *planta* (WRi) and, C) reads of the fungus grown *in* *vitro* (Boiv) (Supplementary Figure 2). Ribosomal RNA contamination was removed from the three files with BBDuk version 38.84 (https://sourceforge.net/projects/bbmap) using the following parameters: a k-mer length of 25 nucleotides, a maximum edit distance of 1, and a reference file of previously characterized ribosomal RNA sequences retrieved from release 132 of the SILVA database (https://www.arb-silva.de). The reads in each pooled library were inspected for removal of low-quality sequences and standard Illumina sequencing adapters contamination with FastQC version 0.11.8. Low-quality bases, adapter contamination, and short reads after trimming were removed with Trimmomatic version 0.33. Quality trimming was performed in 4 bp sliding windows from the 3′ end of the read, removing windows that had a mean base quality score < 15. Reads that were shorter than 18 nucleotides after adapter and quality trimming, were discarded. Summaries of the filtering and trimming are presented in Supplementary Table 2.

Reads within each file WRm, WRi, and Boiv were assembled into “super transcripts” (Davidson et al., 2017) with Trinity version 2.10.0 (Grabherr et al., 2011) following the protocol outlined by Haas et al. (2013) with a K-mer value of 31 bp, *in silico* normalization coverage of 100-fold, and the minimum contig length > 250 bp. The resulting draft transcriptomes were named t_WRm, t_WRi, and t_Boiv (Supplementary Figure 2) producing 86,876; 97,258; and 27,684 super transcripts (hereafter called “transcripts”) with total assembly lengths of 78,356,201 bp; 84,298,903 bp; and 33,981,368 bp, respectively (Supplementary Table 3).

The quality of each transcriptome was assessed with two metrics. First, the proportion of evolutionary informed, expected-single-copy orthologues (gene completeness) represented in the assembly was identified using Benchmarking Universal Single-Copy Orthologs (BUSCO v 4.0.6; Simão et al., 2015). The Liliopsida section within the OrthoDBv10 curated database (Kriventseva, et al., 2019) was used to test t_WRm and t_WRi (prior to separation of plant and fungal transcripts), and the Ascomycota section within the same database, was used for the t_Boiv (Kriventseva et al., 2019). Assemblies were acceptable if they had at least 65% of single-copy orthologs represented completely. Second, the assemblies were tested for RNA seq read representation by aligning the trimmed and filtered reads to the assembled transcriptomes. The mapping was performed with Bowtie2 version 2.3.4.1 (Langmead and Salzberg, 2012), and the alignments were processed with SAMTools version 1.9 (http://www.htslib.org), both using default parameters. Assemblies were of acceptable quality if at least 85% of the source reads mapped to each assembly.

BUSCO confirmed that each plant assembly, t_WRm and t_WRi, before merging into t_WRim, was of high quality with 68.2% and 69.5% single-copy orthologs, respectively. t_Boiv had 90.2% single-copy orthologs (Supplementary Table 4). All of the assemblies have high representation (≥ 90%) of the source RNAseq reads (Supplementary Table 5).
