# Supplementary material for: Interactive transcriptome analyses of Northern Wild Rice (Zizania palustris L.) and Bipolaris oryzae show convoluted communications during the early stages of fungal brown spot development
Source: Front Plant Sci. 2024 Apr 26;15:1350281. doi: 10.3389/fpls.2024.1350281 (PMC11086184; doi:10.3389/fpls.2024.1350281)
Supplement: Supplementary file 25 [file DataSheet_3.docx]

**Supplementary Data 3. Terms used for selection of expressed genes associated to plant defense and to fungal pathogenesis.**

Plant transcripts with one of the broad annotation terms below were selected as candidates: “receptor", "defense", "resistant", "resistance", "disease", "susceptib", "response to fungi", "response to bacterium", "response to stress", "response to wounding”, “interaction", "lysm", "MAPK", "mitogen", "oxidative stress", "ROS", "peroxidase", "catalase", "disease response", "defense pathway", "hypersensitive response", "SNARE", "jasmon", "salicyl", "ethylene", "systemic", "signaling", "transcription factor", "secondary", "phytoalexin","hormon". Pfam domain of protein kinases: "PF00069", "PF07714", "PF08263", "PF00560", "PF12799", "PF13516", "PF13504", "PF13855", "PF00139", "PF00954", "PF01476", "PF13947", "PF14380", "PF00314", "PF01657", "PF00931", "PF13499", "PF03822", "PF00141", and "PF13202".

Some of those terms were cut short to increase the likelihood of capturing more genes of interest. Plant defense genes selection was based on literature (Zipfel, 2009, Zipfel, 2014, Lo Presti et al., 2015, Boutrot and Zipfel, 2017, Buendia et al., 2018; Saijo et al., 2018, Dumanović et al., 2020):

Fungal genes related to pathogenesis were selected mostly based on a joint databases of DGE and the variance partition analysis and included the annotation terms "virulence", "pathogenicity", "pathogenesis", "disease", "necrosis", "effector", "degrading enzymes", "transcription factor", "hydrophobin", polyketide", "non ribosomal", "toxin", "cutinase", "glycosyl hydrolase", "CWDE", "XYL1", "XYL2", "ALP1", "Cel1","Cel2", "XYP1", "HDA1","SNF1", "tentoxin", "cellulase", "hemicellulase", "pectinase", "pectate", "lyase", "cellulose", "hemicellulose", "pectin", "peroxidase", "ROS", "oxidative stress", "thioredoxin", "peroxiredoxin", "superoxide dismutase","xylan", "shikimate", "benzoate", "ethylene", "necrosis inducing peptide", "glycosylation", “SNARE”. In addition, further selections were done based on literature (Wang et al., 2014; Toruño et al., 2016; Franceschetti et al., 2017; Shao et al., 2021). Some of those terms were cut short to increase the likelihood of capturing more genes of interest.
